# The Profiling of Diet and Physical Activity in Reproductive Age Women and Their Association with Body Mass Index

**DOI:** 10.3390/nu14132607

**Published:** 2022-06-23

**Authors:** Mamaru Ayenew Awoke, Thomas P. Wycherley, Arul Earnest, Helen Skouteris, Lisa J. Moran

**Affiliations:** 1 Monash Centre for Health Research and Implementation, School of Public Health and Preventive Medicine, Monash University, Clayton, VIC 3168, Australia; mamaru.awoke@monash.edu; 2 Alliance for Research in Exercise, Nutrition and Activity (ARENA), Allied Health and Human Performance, University of South Australia, Adelaide, SA 5001, Australia; tom.wycherley@unisa.edu.au; 3 Department of Epidemiology and Preventive Medicine, School of Public Health and Preventive Medicine, Monash University, Melbourne, VIC 3004, Australia; arul.earnest@monash.edu; 4 Health and Social Care Unit, School of Public Health and Preventive Medicine, Monash University, Melbourne, VIC 3004, Australia; helen.skouteris@monash.edu; 5 Warwick Business School, Warwick University, Coventry CV4 7AL, UK

**Keywords:** diet, physical activity, body mass index, dietary guideline, reproductive age women

## Abstract

Pre-pregnancy, pregnancy and postpartum are critical life stages associated with higher weight gain and obesity risk. Among these women, the sociodemographic groups at highest risk for suboptimal lifestyle behaviours and core lifestyle components associated with excess adiposity are unclear. This study sought to identify subgroups of women meeting diet/physical activity (PA) recommendations in relation to sociodemographics and assess diet/PA components associated with body mass index (BMI) across these life stages. Cross-sectional data (Australian National Nutrition and Physical Activity Survey 2011–2012) were analysed for pre-pregnancy, pregnant and postpartum women. The majority (63–95%) of women did not meet dietary or PA recommendations at all life stages. Core and discretionary food intake differed by sociodemographic factors. In pre-pregnant women, BMI was inversely associated with higher whole grain intake (β = −1.58, 95% CI −2.96, −0.21; *p* = 0.025) and energy from alcohol (β = −0.08, −0.14, −0.005; *p* = 0.035). In postpartum women, BMI was inversely associated with increased fibre (β = −0.06, 95% CI −0.11, −0.004; *p* = 0.034) and PA (β = −0.002, 95% CI −0.004, −0.001; *p* = 0.013). This highlights the need for targeting whole grains, fibre and PA to prevent obesity across life stages, addressing those most socioeconomically disadvantaged.

## 1. Introduction

Reproductive age women are at higher risk of longitudinal weight gain and developing obesity [1]. Data from longitudinal studies reports that women gain on average up to 0.7 kg per year, and there are greater rates of weight gain in women aged 18–50 years compared to women aged 50 and over [1]. Reproductive life stages, including preconception, pregnancy and postpartum, are critical windows that drive weight gain and maternal adiposity [2]. Nearly 50% of women enter pregnancy with overweight or obesity [3] or gain weight above the Institute of Medicine guidelines’ recommendation during pregnancy [4], and postpartum women retain an extra 0.5–3 kg on average during each pregnancy [5]. Overweight and obesity in preconception and during pregnancy increase the risk of maternal complications and adverse birth outcomes [4,6]. Maternal obesity at conception increases the time to conceive, reduces fertility and increases the risk of future comorbidities, such as type-2 diabetes and cardiovascular diseases, including hypertension [6]. Furthermore, higher pre-pregnancy body mass index (BMI) is a strong predictor of excessive gestational and pregnancy complications [7]. Excessive gestational weight gain additionally drives postpartum weight retention (PPWR), which further increases risks for subsequent pregnancies and exacerbates maternal obesity [8].

Diet and physical activity (PA) are key modifiable risk factors in weight gain and obesity, and optimal diet and regular PA are inversely associated with weight gain and obesity [9,10]. Optimal diet and a higher level of PA can therefore prevent weight gain and obesity [11]. Suboptimal diet and PA have been reported in adults at the population level [12,13]. As a specific high-risk population for weight gain and future obesity, women in pre-pregnancy, during pregnancy and postpartum also have unhealthy dietary patterns and poor diet quality [14]. For example, only 7–10% of pregnant and postpartum women meet population-level recommended intakes of healthy core foods [15,16], and 80% of pregnant women are insufficiently active, which persists into postpartum [16,17,18]. This may be related to barriers such as fatigue or a lack of motivation, and confidence and time. Women may also prioritise family commitments (e.g., parenting or household responsibilities) over their personal lifestyles [19,20]. All these barriers to a healthy lifestyle and sociodemographic factors are potentially associated with increased adiposity in pre-pregnancy, excessive gestational weight gain during pregnancy and PPWR. However, there is limited and conflicting research on sociodemographic factors associated with meeting population-level diet and PA recommendations in women across the reproductive life stages [21].

National guidelines broadly recommend targeting unhealthy diet and sedentary behaviour for management of overweight and obesity in the general population [22]. Women at key reproductive life stages may also benefit from targeting specific diet and PA components to prevent excess adiposity. Identifying both specific diet and PA components and specific groups of reproductive age women could contribute to future interventions for preventing weight again and obesity. This would also contribute to the evidence base for tailoring intervention strategies to improve healthy eating and increase PA in specific high-risk groups of women. We hypothesise that women across reproductive life stages have inadequate diets and PA levels, which may be disparately linked with sociodemographic characteristics, including age, ethnicity, geographic location, marital status, employment and educational and socioeconomic disadvantages. We also hypothesise that higher intakes of specific core foods and increased PA will be associated with lower BMI, but increased total energy intake and higher energy from total discretionary foods will be associated with higher BMI.

The aims of this study were: (ⅰ) to identify women who meet and do not meet diet and PA population-level recommendations based on sociodemographic factors and (ⅱ) to assess the key diet and PA components associated with BMI in women across the reproductive life stages.

## 2. Materials and Methods

### 2.1. Data Source and Study Participants

We used data from the National Nutrition and PA Survey (NNPAS) component of the 2011–2012 Australian Health Survey (AHS) conducted by the Australian Bureau of Statistics (ABS) between May 2011 and June 2012. This national survey was designed to provide detailed information on the health and wellbeing of the Australian population. A stratified multistage sampling of urban and rural private dwellings was obtained to ensure a representative sample of Australians (N = 12,153). Detailed information on participant recruitment, the survey design, data collection and response rates have been previously reported in the Australian Health Survey User Guide [23].

Data were collected using a face-to-face interview from randomly selected people in each selected household (one adult ≥ 18 years and one child aged 2–17 years where applicable). Ethical approval was not required because this study was based on secondary data using Confidentialised Unit Record Files (CURF).

**Figure 1:**
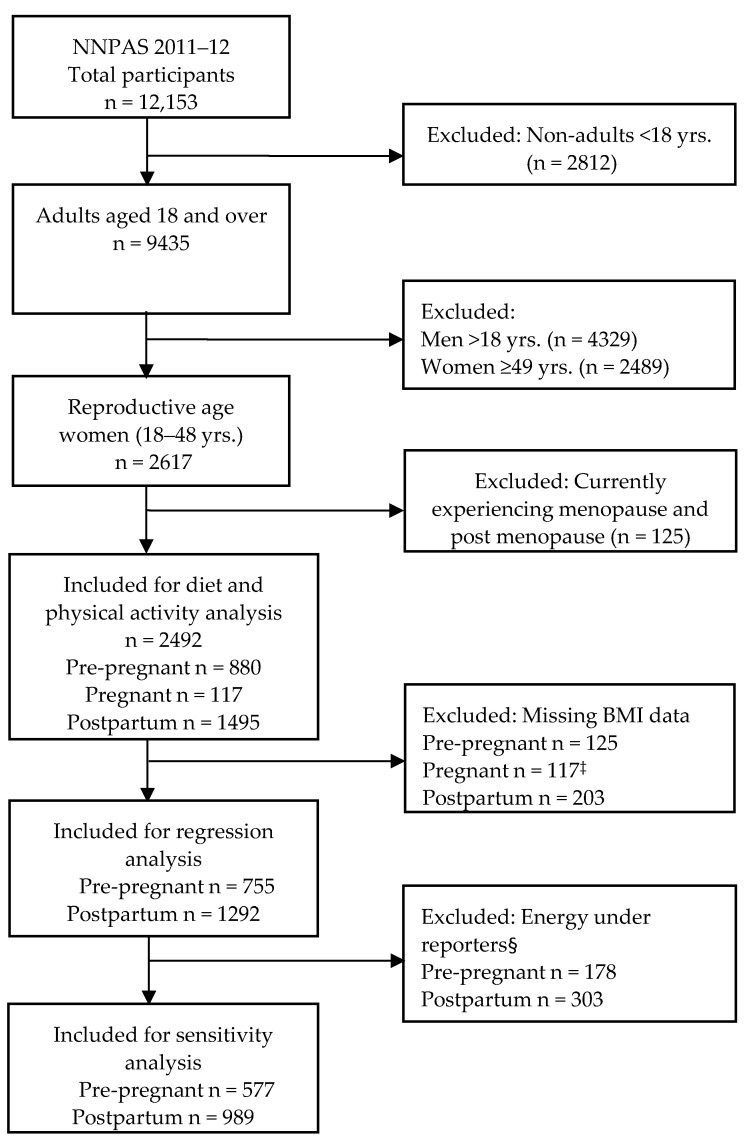
Flow diagram of study participant inclusion for analysis. NNPA, National Nutrition and Physical activity Survey. ‡ Weight/BMI measurement from pregnant women was not taken. § Energy under reporters based on Goldberg cut-off (EI:BMR < 0.9).

This sub-study was a cross-sectional analysis limited to reproductive age women in various key reproductive life stages (pre-pregnancy, pregnancy and postpartum) (Figure 1) (N = 2492). Key reproductive life stages were identified based on proxy questions on ‘female life stages’ and ‘number of children’ in the household (household type, ‘HHTYP’ variable). The question ‘female life stages’ has responses: 1: Have never menstruated. 2: Currently pregnant. 3: Currently breastfeeding. 4: Currently experiencing menopause. 5: Post menopause. 6: None of these apply. 9: Not applicable. To identify pre-pregnancy and postpartum, responses 4 and 5 were excluded. Then, female life stage responses 1 or 6 or 9 (have never menstruated OR none of these apply OR not applicable) AND household type responses: 1 (person living alone), 2 (couple only), 5 (unrelated persons aged 15+ only), 6 (all other households) AND age 18–48 (to exclude lower limit of perimenopause [24]) were classified as pre-pregnancy. Female life stage responses: 3 or 6 or 9 (current breastfeeding OR none of these apply OR not applicable) AND household type responses: 3 (couple family with children) or 4 (one parent family with children) AND age 18–48 were classified as postpartum women; and those who responded currently pregnant taken as pregnant women (Table S1). We note that by this definition, pre-pregnant women were all reproductive age women who were not pregnant or postpartum at the time of the survey.

### 2.2. Variables and Measures

#### 2.2.1. Dietary Assessment

Dietary information was collected face-to-face using 24 h dietary recall administered by trained interviewers. ABS used an Automated Multiple-Pass Method (AMPM) [25] developed by the Agricultural Research Service of the United States Department of Agriculture to capture all foods, beverages and dietary supplements. The nutrients and energy (kJ) intake were calculated from each food and beverage consumed using the 2011–13 Australian Food and Nutrient (AUSNUT) food composition database developed by Food Standards Australia New Zealand (FSANZ). Individual foods were each given an eight-digit food code and classified into food classification groups using the AUSNUT 2011–13 database. Two-day and 24 h dietary recall were collected; the second day was collected via a telephone interview conducted 8 days or more after the first interview. The first day’s dietary recall response rate was 98% (n = 12,153), and the second day’s recall response rate was 64% (n = 7735). The first day of dietary recall was used for all analyses to retain a larger sample size and ensure national representativeness consistent with previous studies [13,26,27].

Daily serves of the five core food groups and total daily energy from discretionary foods/beverages were calculated. Details of the five core food group serving size definitions and daily recommended intake are presented in Table S2. The usual daily intakes of fruit and vegetables (serves per day), grain/cereal foods (serves/day), whole grains (serves/day, g/day, as half proportion of grains), dairy products (serves per day), lean meats and alternatives (serves per day), total energy intake (kJ/d), energy from macronutrients (carbohydrate (%E), protein (%E), total fat (%E), saturated fat (%E), polyunsaturated fat (%E), monounsaturated fat (%E)) and fibre (g/day) were included in the analyses. Discretionary foods and beverages, including percentages of energy from total discretionary foods/beverages, sugar sweetened beverages (SSBs), saturated fats, alcohol intake and added sugar, were included in the analyses. The ABS classified discretionary foods and beverages in the NNPAS using the discretionary flag list, which was based on food grouping level (five-digit code, e.g., 11,501 soft drinks non-cola, 11,503 soft drinks cola) or individual food level (eight-digit codes where the flag is assigned to individual food codes within the five-digit subgroup). A list of discretionary choices and respective food codes with examples are presented in Table S3.

#### 2.2.2. PA

Self-reported PA levels were assessed using the Active Australia Survey, which has been validated against accelerometers in middle-aged women [28]. Respondents reported the estimated time spent in walking, moderate-intensity activity (e.g., gentle swimming, social tennis doubles, golf) and vigorous PA (e.g., jogging, fast cycling, circuit training, competitive tennis) in the past week. The reported durations (excluding the number of sessions) of these activities were summed (sum of minutes) to estimate the total time spent in PA. We used only the duration of PA reported during the previous week to ensure comparability with international guidelines. Total minutes of PA was dichotomised as meeting (≥150 min/week) or not meeting the guidelines (<150 min/week) according to the 2014 Australia’s PA and Sedentary Behaviour Guidelines for Adults [29]. Furthermore, the reported durations for moderate and vigorous activity (multiplied by two) were summed to estimate the total time spent in moderate–vigorous PA (MVPA), which was used both as a continuous variable and dichotomised as ≥150 MVPA minutes/week or ≤ 150 MVPA minutes/week. In multivariable analysis, we included total PA, as it includes all types of activity, such as walking and moderate to vigorous activities, which can be common across life stages.

#### 2.2.3. Covariates

Covariates in the analyses included age (in years), marital status (married vs. not married), country of birth (Australian born, mainly English-speaking country born, other countries), educational level (bachelor/graduate diploma, certificates/advanced diploma or other no non-school qualifications), socio-economic index for areas (SEIFA) or index of relative socio-economic disadvantage (IRSD) (in quintiles: quintile one corresponds to the lowest scores for the most disadvantaged areas and quintile five represents the highest scores for the most advantaged areas), remoteness (inner regional Australia, major cities and other (outer regional/remote)), household income (in quintiles considered as continuous in regression analyses) and health behaviours, such as smoking status (current smoker, ex-smoker or never smoked) and self-assessed health (excellent/very good, good, fair and poor). Participants were also asked whether they were currently on a diet: responses included currently on a diet to lose weight, currently on a diet for health reasons, currently on a diet to lose weight and for health reasons, not currently on a diet or not applicable. Responses were dichotomized to currently on a diet for any reason and not currently on diet.

#### 2.2.4. Dependent Variable

Anthropometric measures (weight and height) of the respondents were taken during the interview using a digital scale (maximum 150 kg and recoded to the nearest 0.1 kg) and a stadiometer (maximum 210 cm and recorded to the nearest 0.1 cm) respectively. Participants were encouraged to remove their shoes and heavy clothing before measurements were taken. Height measurements were repeated on a random 10% sample of respondents to validate the measurement, and if the second measurement of height or waist varied by more than one centimetre, then a third reading was taken. Body mass index (BMI, kg/m^2^) was calculated from measured weight and height as weight in kilograms divided by the square of height in metres. Anthropometric data of women who were pregnant at the time of the survey were not obtained. According to WHO categories, BMI was defined as underweight (<18.5 kg/m^2^), normal weight (18.5–24.9 kg/m^2^), overweight (25.0–29.9 kg/m^2^) and obese (≥30 kg/m^2^).

### 2.3. Statistical Analysis

Descriptive statistics were used to estimate the proportion and mean consumption of dietary intake and PA across reproductive life stages (pre-pregnancy, pregnancy and postpartum). Pearson Chi-square tests were used to determine differences between categorical variables and student’s t-test for continuous variables.

Univariable and multivariable linear regression analyses were performed to investigate diet, PA and sociodemographic factors associated with BMI in pre-pregnancy and postpartum. For multivariable regression, residuals were checked and met the normality assumption. All estimates (proportion, means, standard error, beta-coefficients and 95% CI) were population weighted to take into account sampling weights and sampling design of the survey by applying replicate weights. Jack knife replicate weights were used to obtain unbiased standard errors and coefficient estimates. The analysis was based on complete case data, and codes followed recommendations [30] to account for the complex survey design.

The backward stepwise regression technique was used to select most appropriate variables, removing the least significant variables one by one (variable with the highest *p*-value in the model) and continued until a parsimonious model was reached (*p* < 0.05). The variables were assessed for multicollinearity through the variance inflation factor (VIF) and tolerance statistics (VIF > 10) to exclude the redundant explanatory variables. Collinear variables were excluded, as they showed linear relationship with the other independent variables. All statistical analyses were performed using STATA SE version 16.1 (StataCorp LLC, College Station, TX, USA). Statistical significance was considered at *p*-value ≤ 0.05.

### 2.4. Sensitivity Analysis

Under-reporting is common in nutrition surveys, as people tend to underestimate their food intakes [31], which would affect the overall results. The most utilised method to identify under-reporters is to compare each person’s basal metabolic rate (BMR) with their reported energy intake (EI) and apply Goldberg cut-off values to examine whether the EI reported is plausible. We employed this approach to identify under-reporters, and a sensitivity analysis was performed in women only with plausible energy intakes (excluding under-reporters) consistent with previous studies [32,33]. Briefly, BMR is the amount of energy needed for an individual’s minimum set of body functions required for life over a 24 h period. This was calculated in kilojoules per 24 h based on individual’s age, sex and weight without activity level adjustment. The ratio of energy intake (EI) to BMR (EI:BMR) was used to identify under-reporters (implausibly low energy intakes) using the Goldberg cut-off limit of 0.9 for EI:BMR. This is for data below the 95% confidence limit for an individual, allowing for daily variation in energy intakes and errors in EI:BMR computation. After excluding 481 women (pre-pregnancy n = 178 and postpartum n = 303) with implausibly low energy intake, n = 577 pre-pregnant and n = 989 postpartum women were included for sensitivity analysis in the multivariable model (Figure 1). Approximately a quarter of the total analytical sample, with similar proportions of pre-pregnant (23.6%) and postpartum women (23.5%), were excluded in the sensitivity analysis.

## 3. Results

### 3.1. Sociodemographic Characteristics 

Socio-demographic characteristics across reproductive life stages are presented in Table 1. The mean ages of study participants were 31.2 ± 8.4, 29.3 ± 5.3 and 33.6 ± 8.7 years for pre-pregnant, pregnant and postpartum women, respectively. One in five (20.7%) and 19.3% of pre-pregnant women were overweight and obese, respectively, whereas 25.4% and 22.3% of postpartum women were overweight and obese, respectively. The majority of the participants (64.8% pre-pregnant, 66.7% pregnant and 74.9% postpartum) were Australian born. Next, 42.0% of pre-pregnant, 33.8% of pregnant and 29.4% of postpartum women had high education levels (bachelor’s degree or graduate diploma); 38.6% of pre-pregnant, 69.6% of pregnant and 52.2% of postpartum women were married; 58.6% of prepregnant, 55.6% of pregnant and 60.3% of postpartum women reported never smoking; and 90.0% of pre-pregnant, 96.0% of pregnant and 90.6% of postpartum women reported ‘excellent/very good/good’ self-rated health.

### 3.2. Proportion of Reproductive Age Women Meeting Recommended Intakes of Core Foods, Discretionary Choices and PA

**Figure 2:**
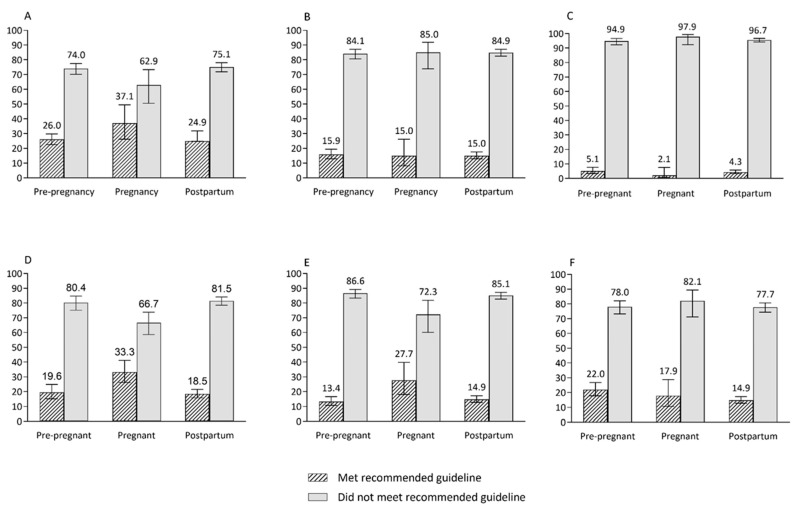
The proportions of women who met and do not meet the population-level recommended intakes of core food groups across reproductive life stages. (**A**) Fruit, (**B**) vegetables and legumes, (**C**) fruit and vegetable combined, (**D**) grain (cereal) foods, (**E**) milk and alternatives, (**F**) meat and alternatives.

The mean intakes of core food groups, discretionary foods, energy from macronutrients and PA across reproductive life stages are shown in Table 2 and Table 3; and the proportions of women who met and did not meet population-level dietary recommendations are shown in Figure 2. Similar mean proportions of total daily energy were from discretionary foods, beverages and SSBs in pre-pregnant (33.4% and 3.77% respectively), pregnant (29.1% and 4.94% respectively) and postpartum (31.5% and 3.51% respectively) women (Table 2).

Approximately one in ten women across all life stages obtained much of their daily energy from added sugars, and this portion was slightly higher in pregnant women (11.8%).

**Figure 3:**
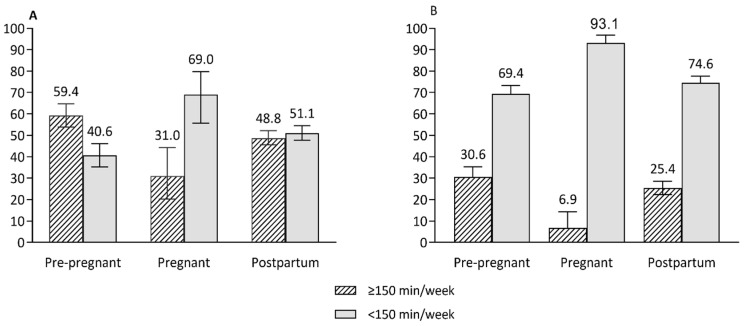
The proportions of women who spent time performing physical activity at least 150 min per week or more across reproductive life stages. (**A**) Total minutes of physical activity per week, (**B**) moderate–vigorous intensity activity per week. Error bars represent 95% CI of proportions.

Despite reproductive age women not meeting the recommended serves of core foods, energy intake from macronutrients was generally in the optimal range across reproductive life stages (Table 3) (acceptable macronutrient distribution range: 15–25% of energy from protein, 20–35% of energy from fat and 45–65% of energy from carbohydrates) [34]. Similar proportions of women met the recommended daily intakes of both fruit and vegetables (5.12% of pre-pregnant, 2.14% of pregnant and 4.35% of postpartum women), vegetables (15.9% for pre-pregnant and 15.0% for pregnant and postpartum women) and meat and alternatives (17.9% of pregnant and 22.0% of pre-pregnant and postpartum women) (Figure 2). The proportions of women who met recommended intakes of fruit, grains/cereals and dairy or alternatives were higher for pregnant women (37.1%, 33.3% and 27.7%, respectively) than pre-pregnant (26.0%, 19.6% and 13.4%, respectively) and postpartum (24.9%, 18.5% and 14.9%, respectively) women (Figure 2). The proportion of women meeting PA guidelines (total activity in minutes) was lower for pregnant women (31.0%) than for pre-pregnant (59.4%) and postpartum (48.8%) women. Similarly, a low proportion of pregnant women (6.9%) had ≥150 min MVPA/week compared to pre-pregnant (30.6%) and postpartum women (25.4%) (Figure 3).

Differences in sociodemographic characteristics for women meeting or not meeting population recommendations for diet and PA pre-pregnancy and postpartum are reported in Tables S4a–g. These data are not presented for pregnant women due to the small sample size. For pre-pregnant women, the recommended intakes of vegetables; fruit; dairy or alternatives; and meat or alternatives, did not differ by sociodemographic factors. For post-partum women, the recommended intakes of dairy or alternatives and meat or alternatives did not differ by sociodemographic factors. Pre-pregnant women born in Australia were less likely to meet the recommended intake of grains/cereals and more likely to have intake of discretionary foods above the recommended level (>2.5 serves/day). Those with a higher education and SEIFA were more likely to meet the PA recommendations. Postpartum women with a higher education were more likely to meet the recommended intakes of vegetables and fruit; those born in Australia were less likely to meet the recommended intakes of fruit and grains/cereals and discretionary foods; those with professional jobs were more likely to meet the recommended intake of fruit, and those with a higher education and SEIFA were more likely to meet the PA recommendations.

### 3.3. Diet and PA Variables Associated with BMI

In multivariable analysis among pre-pregnant women, BMI was inversely associated with higher intake of whole grains (β = −1.58, 95% CI −2.96, −0.21; *p* = 0.025) and with increased energy from alcohol (β = −0.08, 95% CI −0.14, −0.005; *p* = 0.035) (Table 4). However, no associations were found among core foods (fruit, vegetable, grain/cereal foods, dairy, meat and/or alternatives), total energy intake, energy from discretionary foods/beverages, SSBs and PA. With regard to sociodemographic factors, in pre-pregnant women, age (β = 0.22, 95% CI 0.15, 0.29; *p* < 0.001), being born in other county (β = −3.20, 95% CI −4.52, −1.88; *p* < 0.001), being a current smoker (β = −1.40, 95% CI −2.76, −0.04; *p* = 0.044), excellent/very good/good health (β = −2.89, 95% CI −5.51, −0.28; *p* = 0.030) and currently on a diet (β = 2.25, 95% CI 0.25, 4.24; *p* = 0.028) were independently associated with BMI (Table 4).

In postpartum women, BMI was inversely associated with increased fibre intake (β = −0.06, 95% CI −0.11, −0.004; *p* = 0.034) and each minute increase in PA per week (β = −0.002, 95% CI −0.004, −0.001; *p* = 0.013) (Table 5). There were no significant associations between fruit, vegetable, whole grain, dairy, meat and/or alternatives, total energy intake and energy from discretionary foods and BMI in postpartum. Higher socioeconomic disadvantage (β = −1.73,95% CI −3.12, −0.05; *p* = 0.017; Q5 vs. Q1) and excellent/very good/good health condition (β = −2.30, 95% CI −4.06, −0.54; *p* = 0.011) were inversely associated with BMI, whereas currently on diet (β = 2.82, 95% CI 1.76, 3.89; *p* < 0.001) and increased age (β = 0.13, 95% CI 0.08, 0.19; *p* < 0.001) were positively associated with BMI (Table 5).

### 3.4. Results from Sensitivity Analysis 

Multivariable linear regression analysis results of diet and PA and BMI after excluding implausible energy reporters in pre-pregnant and postpartum women are presented in Table 6. In pre-pregnant women, the associations between whole grains and energy from alcohol and BMI were not maintained in the sensitivity analysis. Other diet variables showed similar associations in terms of directionality without substantial differences in the magnitudes of estimates (<20% relative change) from the main analysis. In postpartum women, the association between fibre and BMI was no longer statistically significant, but the total energy intake’s association with BMI became statistically significant. The inverse association between increased PA and BMI was maintained after excluding implausible energy reporters.

## 4. Discussion

### 4.1. Main Findings

We report here for the first time on diet and PA and their associations with BMI in a nationally representative sample of Australian women across key reproductive life stages. We confirm women across life stages failed, on average, to meet population-level recommended intakes of key core foods. A higher proportion of daily energy from discretionary foods persisted in pre-pregnant, pregnant and postpartum women. Sociodemographic factors, including country of birth, education, occupation and socioeconomic disadvantage areas, were associated with core and discretionary food intake and PA in pre-pregnancy and postpartum women. An inverse association was observed for both higher whole grain intake and higher energy from alcohol and BMI in pre-pregnant women, whereas increased fibre intake and PA were inversely associated with BMI in postpartum women.

### 4.2. Meeting Recommended Intakes of Core Foods, Discretionary Choices and PA

Our findings of failure to meet population-level recommendations for core foods, discretionary foods and PA are consistent with previous studies in pre-pregnant, pregnant [21,36] and postpartum women [37,38] and in the general population [13]. Failure to meet dietary or PA recommendations may be explained by factors, including lack of awareness, limited resources for accessing healthy foods for low-socioeconomic-status women, [36], cultural influences on food choice, lack of social support, exposure to fast food outlets [39] and other psychosocial barriers [40]. Low proportions of pregnant and postpartum women met the PA guidelines, which is consistent with previous studies [41,42,43]. Several barriers may prevent pregnant women from engaging in PA, including perceived mother–baby safety concerns, fatigue, lack of motivation and lack of social support [44,45]; and barriers such as time limitations, lack of childcare, lack of partner support and family responsibilities may prevent PA by postpartum women [20]. 

### 4.3. Socioeconomic Factors

Consistent with prior research in the general population [46,47,48], a range of sociodemographic characteristics were associated with meeting the recommended intakes of PA guidelines in pre-pregnant and postpartum women. For women pre-pregnancy, those born in Australia were less likely to have an optimal intake of grains/cereals, and those with higher education and the least socioeconomically disadvantaged were more likely to meet PA guidelines. Conversely, the recommended intakes of vegetables, fruit, dairy or alternatives and meat or alternatives did not differ by sociodemographic factors in pre-pregnant women, as previously reported [21]. Postpartum women with higher education and living in socioeconomically advantaged areas were more likely to have the recommended intakes of vegetables and fruits and the recommended level of PA. While research is limited in postpartum women, this finding is consistent with previous studies reporting socio-economic disadvantage as being a strong determinant of fruit and vegetable intake [46] and PA [48] in the general population. A disparity in overall food and nutrient intake has been previously reported between Australian-born and overseas-born women, with overseas born women having higher intakes of cereals/beans but less vegetable/legume, dairy and meat intakes than Australian-born women [49]. This suggests that future interventions could potentially target grains/cereals and PA for pregnant women; and vegetables, fruit, discretionary foods and PA for postpartum women, specifically those from different ethnic and socioeconomic backgrounds.

### 4.4. Dietary Components and BMI

A higher whole grain intake (≥3 servings/day) in pre-pregnant women and increased fibre intake in postpartum were associated with decreased BMI (kg/m^2^). The association between wholegrains and BMI in pre-pregnancy is consistent with previous meta-analyses in the general population reporting ≥3 servings/day whole grains was associated with a lower BMI and less central adiposity [50]. In postpartum women, a 1 g/d increase in fibre was associated with a 0.06 kg/m^2^ lower BMI and 0.15 kg lower postpartum weight gain [51]; and fibre intake below the recommendation (<29 g/day) increased the risk of PPWR by 24% [52]. These findings may be related to the effects of whole grains [53] and fibre [54] on satiety and fullness and the subsequent inhibitory effect on energy intake. Given the mean wholegrains and fibre intakes were ~1.2 serves/day and ~20 g/day, respectively (compared to broad guidelines of ≥3 serves/day [55] and population recommendations of 25–30 g/day [34], respectively), it is imperative to target both fibre and wholegrains for optimising weight management in women at key reproductive life-stages.

We report an inverse association between BMI and energy from alcohol in pre-pregnant women. While there is a lack of research currently on the association between alcohol and obesity in pre-pregnant and postpartum women, there are inconsistent findings in the general population [56,57,58,59]. This may be partly attributed to variations in frequency, amount or types of alcohol, and variations in lifestyle and dietary habits or energy intake for drinkers and non-drinkers [60]. Our analysis was adjusted for factors such as dieting and total energy intake, and indicates an independent relationship between alcohol and BMI in pre-pregnant women. The link between alcohol intake and BMI is likely complex and modifiable across life stages due to physiological variations. Furthermore, given that a large proportion of pregnancies are unplanned [61] and population recommendations are to stop alcohol intake when trying to conceive [35], the contribution of alcohol to both BMI and adverse pregnancy outcomes must be considered.

We observed no significant association between fruit or vegetable intake and BMI in pre-pregnant and postpartum women. This is in contrast to prior studies reporting inverse associations between the ‘vegetables and meat’ pattern and BMI in preconception [14] and the fruit and vegetable index and weight gain in young women [62], and systematic reviews reporting an inverse association between fruit and vegetable intake and BMI in the general population [63]. This discrepancy could be due to the lack of consistent adjustment for important confounders, including total energy intake and the low intake of fruit and vegetables. In contrast to reports in the general population [64,65], we also report no association between energy from discretionary foods or SSBs and BMI in pre-pregnant and postpartum women. These disparate results are unclear, but may be due to the use of different analysis approaches. We used energy from discretionary foods, unlike prior studies that assessed individual discretionary foods [65]. Total energy intake was not also associated with BMI in pre-pregnant and postpartum women, in contrast to prior studies of postpartum women [66]. This may be partly explained by energy misreporting, particularly in women with higher BMIs [67], or other factors, such as the relatively high rate of dieting. In sensitivity analysis, however, increased energy intake was associated with BMI in postpartum women, even after exclusion of energy misreporters. Here, for our main analysis, we reported results without exclusion of energy misreporters, as these are consistent with prior reports from this large national survey [68].

### 4.5. PA and BMI

We report here a modest but significant inverse association between PA and BMI in postpartum women, which is consistent with some [69,70] but not all [71] observational studies. Conversely, we found no significant association between PA and BMI in pre-pregnant women, which is in contrast to longitudinal studies in reproductive-aged women that reported an inverse association between a higher level of MVPA and weight gain [72] or overweight and obesity [73]. However, these studies did not consistently adjust for important confounders, such as energy intake and other dietary factors. Differences in study design (cross-sectional vs. longitudinal) may partly explain the inconsistent reports. It is difficult to explain the finding here that PA is more closely associated with BMI only in postpartum women, but this may be related to the smaller sample size for the pre-pregnant population. Given over half of women currently undertake suboptimal amounts of PA [16] and the benefits of PA for psychological and physical wellbeing [74] and weight management [69], there is a need for further research to elucidate the mixed findings of PA and BMI in free-living pre-pregnant and postpartum women.

### 4.6. Strength and Limitations

This study has several strengths. Given the use of a subsample from a nationally representative survey, the results can be generalisable to the Australian population of reproductive age women. Height and weight were collected based on measured data, which give accurate BMI status and more reliable estimates than self-reported data. Furthermore, our analysis followed rigorous methods by accounting for sampling weight and survey design, resulting in unbiased estimates. The analysis also adjusted for several important confounders, such as dieting. However, the limitations of this study should be acknowledged. First, self-reported dietary data based on 24 h recall may be subject to recall bias or misreporting due to social desirability bias [75], which affects the results towards the null. However, the use of AMPM aids to minimize recall bias by maximising the recall of foods and accounting for intrapersonal variability [25]. Second, although 24 h recall gives a good estimation of dietary intake at the population level, dietary data based on one day recall may not reflect the usual intake of foods and nutrients. Third, we used a proxy method to identify reproductive life stages using prespecified terms ‘female life stages’ and ‘number of children’ in the household that may not definitely separate pre-pregnancy and postpartum women. Despite the lack of certainty on the definitions of the specific reproductive life stages, this dataset allowed the use of robust methods of dietary assessment by 24 h recall. Fourth, the sample size for pre-pregnant and pregnant women was relatively small, which may have reduced the power in the multivariable analysis for pre-pregnant women. Finally, the cross-sectional study design precluded the assessment of any causal relationships and may also explain inconsistencies with previous longitudinal studies. We also note that some food group recommendations are different for lactating women. We did not differentiate postpartum women based on breastfeeding status, as the subgroup sample sizes, particularly for sociodemographic analysis, would have been too small.

## 5. Conclusions

Our findings showed that women across reproductive life stages, in particular, those from lower socioeconomic groups and those born in Australia, failed to meet population-level diet and PA recommendations. In pre-pregnant women, whole grains and energy from alcohol were inversely associated with BMI, and fibre and PA were associated with BMI in postpartum women. This study suggests that the lifestyle components of whole grains, fibre, alcohol and PA; and sociodemographic groups of country of birth, education and socioeconomic disadvantage, should be targeted in future interventions to prevent weight gain or obesity in women across reproductive life stages. The findings, however, should be interpreted with caution due to the indirect definitions of reproductive life stages.

## Figures and Tables

**Table 1:** Sociodemographic characteristics across reproductive life stages (n = 2492).

	Pre-Pregnancy (n = 880)	Pregnancy (n = 117)	Postpartum (n = 1495)
	n (%) ^a^	n (%) ^a^	n (%) ^a^
**Age, mean (SD)**	31.2 ± 8.4	29.3 ± 5.3	33.6 ± 8.7
**BMI, kg/m^2^**	25.4 ± 6.21	NA	26.2 ± 5.61
**BMI (WHO categories)**			
Under/normal weight (<25 kg/m^2^)	412 (60.3)	NA	639 (52.3)
Overweight (25–<30 kg/m^2^)	180 (20.7)	NA	336 (25.4)
Obese (≥30 kg/m^2^)	163 (19.3	NA	317 (22.3)
**Dieting**			
Not currently on diet	132 (13.6)	1 (0.18)	242 (16.6)
Currently on diet	748 (86.4)	116 (99.8)	1253 (85.1)
**Country of birth**			
Australia	645 (64.8)	87 (66.7)	1113 (74.9)
English speaking countries	80 (10.7)	7 (9.7)	145 (9.8)
Others	155 (24.5)	23 (15.3)	237 (15.3)
**Remoteness area**			
Major cities	596 (79.4)	67 (61.7)	972 (72.6)
Inner regional	150 (14.1)	26 (22.4)	286 (18.2)
Other	134 (6.50)	24 (15.9)	237 (9.16)
**Marital status**			
Married	313 (38.6)	83 (69.6)	880 (52.2)
Not married	567 (61.4)	34 (30.4)	615 (47.8)
**Non-school educational level**			
Bachelor/Graduate diploma	362 (42.0)	41 (33.8)	447 (29.4)
Certificates/Advanced diploma	276 (33.3)	37 (31.9)	523 (35.7)
No non-school qualification	232 (24.7)	39 (34.3)	514 (35.0)
**Household income ^b^**			
Q1 (lowest)	72 (8.21)	17 (17.4)	260 (14.0)
Q2	97 (15.2)	15 (13.0)	265 (17.2)
Q3	127 (17.0)	25 (25.4)	297 (21.9)
Q4	242 (28.3)	27 (23.3)	301 (24.0)
Q5 (highest)	261 (31.4)	28 (20.9)	216 (22.8)
**Occupation**			
Professional	320 (34.2)	37 (29.6)	369 (26.3)
Assoc. Professional	296 (33.0)	30 (24.0)	474 (33.8)
Clerical trade	135 (16.2)	7 (7.94)	177 (13.6)
Other	129 (16.5)	40 (38.5)	475 (26.3)
**SEIFA quintile**			
Q1 (lowest)	151 (15.0)	18 (18.6)	277 (18.6)
Q2	162 (18.4)	36 (25.2)	274 (17.6)
Q3	176 (23.7)	20 (14.7)	316 (21.6)
Q4	158 (20.5)	21 (26.0)	260 (16.9)
Q5 (highest)	233 (22.3)	22 (15.4)	368 (25.4)
**Smoking status**			
Current smoker	201 (23.3)	12 (9.5)	295 (16.3)
Ex-smoker	169 (18.2)	42 (35.0)	380 (23.8)
Never smoked	510 (58.6)	68 (55.6)	820 (60.3)
**Self-assessed health**			
Excellent/very good/good	789 (90.0)	111 (96.0)	1341 (90.6)
Fair/poor	91 (10.0)	6 (4.0)	154 (9.4)

^a^ Weighted percentage (replicate weight accounted); BMI, body mass index; NA, not applicable as BMI data were not collected from pregnant women. SEIFA, socio-economic index of disadvantage; quintile one represents the most disadvantaged areas, and quintile five represents the least disadvantaged areas (higher quintiles correspond to areas with lower levels of disadvantage areas where fewer individuals have low incomes, low educational attainment or work in unskilled occupations); ^b^ Equivalised household income (weekly, AUD)—an indicator of the economic resources available to each member of a household to indicate the situation of individuals and households.

**Table 2:** Core food groups and energy from discretionary foods in women across reproductive life stages.

	Pre-Pregnancy (n = 880)	Pregnancy (n = 117)	Postpartum (n = 1495)	Population Level Recommendations
	Mean ± SD	Mean ± SD	Mean ± SD	
Vegetables, legumes/beans (serve/day)	2.90 ± 2.67	2.95 ± 2.97	2.95 ± 2.97	≥5 serves/day
Fruit (serves/day)	1.32 ± 1.58	1.77 ± 1.88	1.31 ± 1.51	≥2 serves/day
Grain/cereals foods (serve/day)	3.97 ± 2.75	5.21 ± 3.34	4.01 ± 2.67	≥6 serves/day
Milk, yoghurt, cheese and alternatives (serve/day)	1.32 ± 1.11	1.97 ± 1.80	1.39 ± 1.13	≥2.5 serves/day
Meats and alternatives (serve/day)	1.56 ± 1.45	1.24 ± 1.12	1.60 ± 1.46	≥2.5 serves/day
Whole grains (serves/day)	1.26 ± 1.57	1.61 ± 1.80	1.19 ± 1.55	≥3 serves/day
Fibre (g/day)	20 ± 11.20	23.3 ± 12.4	20.5 ± 10.9	≥25 g/day
DF (%E)	33.4 ± 22.4	29.1 ± 18.9	31.5 ± 19.4	<2.5 serves/day
SSBs (%E)	3.77 ± 7.21	4.94 ± 9.70	3.51 ± 6.81	<2.5 serves/day

Proportion is weighted (population weight and survey design accounted); DF, discretionary foods; SSBs, sugar sweetened beverages; %E, percentage energy.

**Table 3:** Energy and macronutrient intake and PA in women across reproductive life stages.

	Pre-Pregnancy (n = 880)	Pregnancy (n = 117)	Postpartum (n = 1495)	Population Level Recommendations
	Mean ± SD	Mean ± SD	Mean ± SD	
Total energy (kJ)	7781.4 ± 3118.6	8683.3 ± 4037.6	7637.2 ± 2880.8	8700 kJ
Protein (%E)	17.8 ± 6.71	17.0 ± 5.48	18.3 ± 5.76	15–25%
CHO (%E)	44.4 ± 11.2	49.4 ± 10.8	44.4 ± 10.7	45–65%
Total fat intake (%E)	31.0 ± 9.14	30.6 ± 8.32	31.8 ± 8.79	20–35%
Saturated and trans-fat (%E)	12.1 ± 4.88	12.3 ± 4.62	12.5 ± 4.74	<10%
Trans-fat intake (%E)	0.56 ± 0.36	0.59 ± 0.34	0.57 ± 0.36	-
Monosaturated fat intake (%E)	11.8 ± 4.20	11.3 ± 3.48	12.2 ± 4.14	-
Added sugar (%E)	10.3 ± 8.92	11.8 ± 11.44	9.44 ± 7.79	<10%
Sodium (mg/day)	2249 ± 1334.0	2295.5 ± 1190.8	2222 ± 1153.2	2000 mg/day ^a^
Alcohol (%E)	3.92 ± 9.61	0.07 ± 0.83	2.49 ± 6.46	<10 standard drink/week ^b^
Total PA (min/week) ^1^	255.4 ± 253.1	149.2 ± 204.6	201.8 ± 224.8	≥150 min/day
MVPA (min/week) ^2^	143.9 ± 248.0	30.6 ± 80.1	115.2 ± 210.7	≥150 min/day

Proportion is weighted (population weight and survey design accounted); CHO, carbohydrates; MVPA, moderate–vigorous physical activity; PA, physical activity; %E, percentage energy.^1^ Total minutes of physical activity undertaken in last week (includes walking for transport + walking for fitness + moderate + vigorous time but does not include sessions). ^2^ Moderate–vigorous physical activity derived from time spent in moderate and vigorous intensity activities (moderate time + 2 times vigorous time). ^a^ Reference for sodium guideline [34]. ^b^ Reference for alcohol guideline [35].

**Table 4:** Associations between diet and physical activity and BMI in pre-pregnant women (N = 755).

	Unadjusted Model		Adjusted Model	
	β (95% CI)	*p* Value	β (95% CI)	*p* Value
**Age, year**	0.20 (0.14, 0.25)	<0.001	0.22 (0.15, 0.29)	**<0.001**
**Country of birth**				
Australia (ref.)				
Other English-speaking country	−0.20 (−2.05, 1.66)	0.832	0.34 (−1.10, 1.79)	0.639
Others	−3.04 (−4.11, −1.96)	<0.001	−3.20 (−4.52, −1.88)	**<0.001**
**Remoteness area**				
Major cities (ref.)				
Inner regional	1.77 (0.22, 3.32)	0.026	0.36 (−1.04, 1.76)	0.613
Other	3.01 (0.83, 5.20)	0.008	1.73 (−0.37, 3.83)	0.104
**Marital status**				
Not married (ref.)				
Married	0.30 (−0.95, 1.55)	0.633	−0.51 (−1.79, 0.77)	0.432
**Education**				
Bachelor/Graduate diploma (ref.)				
Certificates/Advanced diploma	0.99 (−0.42, 2.41)	0.164	−0.97 (−2.62, 0.67)	0.242
No non-school qualification	2.09 (0.34, 3.85)	0.020	−0.56 (−2.18,1.06)	0.491
**Household income (cont. decile)**	−0.08 (−0.33, 0.18)	0.553	··	··
**Occupation**				
Clerical trade (ref.)				
Professional	−0.82 (−2.54, 0.91)	0.347	−0.77 (−2.33, 0.79)	0.327
Assoc. Professional	−0.90 (−2.50, 0.70)	0.265	−1.08 (−2.55, 0.40)	0.150
Other	0.05 (−2.18, 2.27)	0.967	−0.54 (−2.50, 1.42)	0.584
**SEIFA**				
1st (highest disadvantage) (ref.)				
2nd quintile	−0.52 (−2.36,1.32)	0.575	−0.65 (−2.65, 1.36)	0.521
3rd quintile	−2.10 (−3.98, −0.22)	0.029	−1.16 (−2.90, 0.57)	0.184
4th quintile	−1.55 (−3.79,0.69)	0.171	−1.48 (−3.53, 0.57)	0.15
5th quintile (least disadvantage)	−2.18 (−3.98, −0.38)	0.018	−1.85 (−3.71, 0.01)	0.051
**Smoking status**				
Never smoked (ref.)				
Current smoker	0.55 (−1.09, 2.18)	0.505	−1.40 (−2.76, −0.04)	**0.044**
Ex-smoker	1.73 (0.05, 3.41)	0.043	0.53 (−1.08, 2.15)	0.512
**Self-assessed health**				
Fair/poor (ref.)				
Excellent/very good/good	−2.77 (−5.57, 0.03)	0.052	−2.89 (−5.51, −0.28)	**0.030**
**Dieting**				
Not currently on diet (ref)				
Currently on diet	2.41 (0.21, 4.61)	0.032	2.25 (0.25, 4.24)	**0.028**
**Vegetables, legumes/beans (categorical)**				
<1 serves/day (ref.)				
≥1 to <3 serves/day	−0.43 (−1.82, 0.96)	0.537	··	··
≥3 to <5 serves/day	0.49 (−1.24, 2.21)	0.574	··	··
≥5 serves/day	−1.56 (−3.32, 0.21)	0.083	··	··
**Vegetables, legumes/beans (binary)**				
<5 serves/day (ref.)				
≥5 serves/day	−1.48 (−2.93, −0.03)	0.046	−1.06 (−2.54, 0.42)	0.156
**Fruit (categorical)**				
<1 serves/day (ref.)				
≥1 to <2 serves/day	−1.55 (−3.86, 0.72)	0.007	··	··
≥2 to <3 serves/day	−0.79 (−2.50, 0.93)	0.363	··	··
≥3 serves/day	−1.13 (−3.19, 0.94)	0.279	··	··
**Fruit (binary)**				
<2 serves/day (ref.)				
≥2 serves/day	−0.47 (−1.84, 0.90)	0.494	0.39 (−1.04, 1.81)	0.588
**Grain (cereal) foods (categorical)**				
Zero or none (ref.)				
>0 to <2 serves/day	−1.62 (−4.71, 1.47)	0.299	··	··
≥2 to <4 serves/day	−2.48 (−5.67, 0.71)	0.125	··	··
≥4 to <6 serves/day	−2.42 (−5.73, 0.89)	0.149	··	··
≥6 serves/day	−2.78 (−6.09, 0.53)	0.098	··	··
**Grain (cereal) foods (binary)**				
<6 serves/day (ref.)				
≥6 serves/day	−0.64 (−2.07, 0.79)	0.373	0.74 (−0.92, 2.39)	0.376
**Whole grain (categorical)**				
<1 serves/day (ref.)				
≥1 to <2 serves/day	−0.64 (−2.34, 1.07)	0.459	−0.12 (−1.69, 1.44)	0.874
≥2 to <3 serves/day	−1.88 (3.43, −0.33)	0.019	−1.63 (−3.44, 0.17)	0.075
≥3 serves/day	−2.10 (−3.78, −0.41)	0.016	−1.58 (−2.96, −0.21)	**0.025**
**Whole grains (binary)**				
<48 g/day (ref.)				
≥48 g/day	−1.65 (−2.96, −0.34)	0.014	··	··
**Whole grain (half of total grain intake)**				
<50% (ref.)				
≥50%	−1.84 (−3.12, −0.56)	0.006	··	··
**Milk and/or alternatives (categorical)**				
<0.5 serves/day (ref.)				
≥0.5 to <1.5 serves/day	−1.23 (−3.19, 0.73)	0.214	··	··
≥1.5 to <2.5 serves/day	−1.08 (−3.05, 0.89)	0.280	··	··
≥2.5 serves/day	−1.66 (−4.22, 0.89)	0.199	··	··
**Milk and/or alternatives (binary)**				
<2.5 serves/day (ref.)				
≥2.5 serves/day	−0.86 (−2.89, 1.17)	0.401	··	··
**Meats and/or alternatives (categorical)**				
<0.5 serves/day (ref.)				
≥0.5 to <1.5 serves/day	−1.86 (−3.54, −0.17)	0.032	··	··
≥1.5 to <2.5 serves/day	−0.38 (−1.93, 1.17)	0.624	··	··
≥2.5 serves/day	−0.58 (−2.32. 1.17)	0.510	··	··
**Meats and/or alternatives (binary)**				
<2.5 serves/day (ref.)				
≥2.5 serves/day	0.22 (−1.12, 1.55)	0.748	0.87 (−0.34, 2.08)	0.157
**Fibre (g/day)**	−0.07 (−0.11, −0.03)	0.001	··	··
**CHO (%E)**	0.01 (−0.04, 0.07)	0.639	··	··
**Protein (%E)**	0.11 (0.03, 0.20)	0.010	··	··
**Total fat (%E)**	−0.03 (−0.07, 0.02)	0.197	−0.06 (−0.14, 0.03)	0.179
**Trans-fat (%E)**	0.84 (−0.66, 2.34)	0.268	··	··
**Saturated and trans-fat (%E)**	−0.05 (−0.17, 0.07)	0.414	··	··
**Monosaturated fat (%E)**	−0.04 (−0.18, 0.10)	0.545	··	··
**Polyunsaturated fat (%E)**	−0.14 (−0.47, 0.18)	0.381	··	··
**Alcohol (%E)**	−0.03 (0.09, 0.03)	0.352	−0.08 (−0.14, −0.005)	**0.035**
**DF (%E)**	0.02 (−0.01, 0.05)	0.221	0.01 (−0.03,0.05)	0.475
**SSBs (%E)**	0.09 (−0.007,0.18)	0.069	0.04 (−0.06, 0.15)	0.378
**Added sugar intake (%E)**	0.05 (−0.03, 0.13)	0.211	··	··
**Total energy (MJ)**	−0.24 (−0.42, −0.05)	0.013	−0.16 (−0.37, 0.05)	0.135
**Total PA (minutes/wk) cont ^1^**	−0.003 (−0.004, −0.001)	0.005	−0.002 (−0.004, 0.0002)	0.080
**Total PA (minutes/wk) ^1^**				
Did not meet recommended guideline (ref.)				
Met recommended guidelines	−1.38 (−2.86, 0.09)	0.066	··	··

Data were analysed using linear regression with data presented from univariable and multivariable linear regression analyses. Collinear diet variables (VIF > 10) were excluded (protein correlated with meat and alternatives, carbohydrate with alcohol, monosaturated fat, polyunsaturated fat, trans-fat saturated fat + trans-fat correlated with total fat, added sugar correlated with SSBs). β, beta-coefficient; CHO, carbohydrate; DF, discretionary foods; MJ, megajoules; MVPA, moderate–vigorous physical activity; PA, physical activity; SEIFA, socio-economic index of disadvantage for areas; SSBs, sugar sweetened beverage, %E, percentage energy. ^1^ Total minutes of physical activity undertaken in last week (includes walking for transport + walking for fitness + Moderate + Vigorous time but does not include sessions). Used as continuous and dichotomized as whether physical activity last week met 150 min recommended guidelines.

**Table 5:** Associations between diet and physical activity and BMI in postpartum women (N = 1292).

	Unadjusted Model		Adjusted Model	
	β (95% CI)	*p* Value	β (95% CI)	*p* Value
**Age, year**	0.12 (0.07, 0.16)	<0.001	0.13(0.08, 0.19)	**<0.001**
**Country of birth**				
Australia (ref.)				
Other English-speaking country	0.18 (−1.28, 1.65)	0.802	−0.01(−1.59, 1.58)	0.993
Others	−0.45 (−1.67, 0.77)	0.461	−0.98 (−2.31, 0.36)	0.148
**Remoteness**				
Major cities (ref.)				
Inner regional	1.24 (0.07, 2.40)	0.038	0.40 (−0.77, 1.58)	0.496
Other	1.87 (0.45, 3.28)	0.011	1.16 (−37, 3.83)	0.129
**Marital status**				
Not married (ref.)				
Married	0.77 (−0.15, 1.70)	0.101	··	··
**Education**				
Bachelor/Graduate diploma (ref.)	1.36 (0.18, 2.55)	0.025	−0.40 (−1.58, 0.78)	0.500
Certificates/Advanced diploma	0.50 (−0.61, 1.61)	0.373	0.83 (−0.20, 1.87)	0.491
No non-school qualification				
**Household income (cont. decile)**	−0.03 (−0.20, 0.14)	0.703	··	··
**Occupation**				
Clerical trade (ref.)				
Professional	−0.59 (−2.25, 1.07)	0.482	−0.58 (−2.34, 1.18)	0.511
Assoc. Professional	−0.51 (−2.23, 1.21)	0.559	−0.92 (−2.40, 0.55)	0.216
Other	−0.23 (−2.08, 1.63)	0.807	−0.19 (−1.89, 1.51)	0.828
**SEIFA**				
1st (highest disadvantage) (ref.)				
2nd quintile	−0.76 (−2.49,0.97)	0.383	−0.37 (−1.99, 1.24)	0.644
3rd quintile	−1.36 (−3.46, 0.74)	0.199	−1.07 (−3.06, 0.92)	0.286
4th quintile	−1.94 (−3.67, −0.22)	0.028	−1.62 (−3.28, 0.05)	0.058
5th quintile (least disadvantage)	2.30 (−3.75, −0.86)	0.002	−1.73 (−3.12, −0.32)	**0.017**
**Smoking status**				
Never smoked (ref.)				
Current smoker	1.22 (0.20, 2.25)	0.020	0.15 (−0.84, 1.15)	0.761
Ex-smoker	1.49 (0.55, 2.43)	0.002	0.85 (−0.14, 1.84)	0.092
**Self-assessed health**				
Fair/poor (ref.)				
Excellent/very good/good	−3.16 (−4.92, −1.41)	0.001	−2.30 (−4.06, −0.54)	**0.011**
**Dieting**				
Not currently on diet (ref)				
Currently on diet	2.81 (1.75, 3.88)	<0.001	2.82 (1.76, 3.89)	**<0.001**
**Vegetables, legumes (categorical)**				
<1 serves/day (ref.)				
≥1 to <3 serves/day	−0.59 (−1.76, 0.57)	0.311	··	··
≥3 to <5 serves/day	−0.90 (−2.33, 0.53)	0.211	··	··
≥5 serves/day	−0.85 (−2.31, 0.60)	0.246	··	··
**Vegetables, legumes/beans (binary)**				
<5 serves/day (ref.)				
≥5 serves/day	−0.36 (−1.68, 0.96)	0.587	1.04 (−0.53, 2.62)	0.189
**Fruit (categorical)**				
<1 serves/day(ref.)				
≥1 to <2 serves/day	0.73 (−0.40, 1.86)	0.201	··	··
≥2 to <3 serves/day	−0.24 (−1.65, 1.16)	0.731	··	··
≥3 serves/day	−1.49 (−2.94, −0.05)	0.043	··	··
**Fruit (binary)**				
<2 serves/day (ref.)				
≥2 serves/day	−1.09 (−2.07, 0.11)	0.030	−0.29 (−1.44, 0.86)	0.617
**Grain (cereal) foods (categorical)**				
Zero or none (ref.)				
>0 to <2 serves/day	−0.95 (−3.40, 1.51)	0.442	··	··
≥2 to <4 serves/day	−1.51 (−3.561, 0.49)	0.137	··	··
≥4 to <6 serves/day	−1.36 (−3.53, 0.80)	0.212	··	··
≥6 serves/day	−1.93 (−4.14, 0.28)	0.086		
**Grain (cereal) foods (binary)**				
<6 serves (ref.)				
≥6 serves	−0.66 (−1.72, 0.41)	0.222	··	··
**Whole grain (categorical)**				
<1 serves/day (ref.)				
≥1 to <2 serves/day	−1.34 (−2.35, −0.31)	0.459	−0.85 (−1.86, 0.16)	0.097
≥2 to <3 serves/day	−0.52 (−1.91, 0.87)	0.460	−0.40 (−1.80, 1.00)	0.569
≥3 serves/day	−1.90 (−3.11, −0.69)	0.003	−1.08 (−2.54, 0.38)	0.144
**Whole grain (binary)**				
<48 g/day (ref.)				
≥48 g/day	−0.81 (−1.69, 0.73)	0.071	··	··
**Whole grain (half of total grains intake)**				
<50% (ref.)				
≥50%	−0.95 (−1.99, 0.09)	0.072	··	··
**Milk and/or alternatives (categorical)**				
<0.5 serves/day (ref.)				
≥0.5 to <1.5 serves/day	0.64 (−0.40, 1.68)	0.221	··	··
≥1.5 to <2.5 serves/day	−0.16 (−1.46, 1.14)	0.808	··	··
≥2.5 serves	0.04 (−1.24, 1.32)	0.953	··	··
**Milk and/or alternatives (binary)**				
<2.5 serves/day (ref.)				
≥2.5 serves/day	−0.20 (−1.28, 0.88)	0.715	−0.48 (−1.53, 0.57)	0.366
**Meats and/or alternatives (categorical)**				
<0.5 serves/day (ref.)				
≥0.5 to <1.5 serves/day	−0.96 (−2.01, 0.09)	0.071	··	··
≥1.5 to <2.5 serves/day	−0.69 (−1.82, 0.44)	0.228	··	··
≥2.5 serves/day	−0.62 (−1.85, 0.62)	0.322	··	··
**Meats and alternatives (binary)**				
<2.5 serves/day (ref.)				
≥2.5 serves/day	−0.08 (−1.12,0.97)	0.880	−0.29 (−1.48, 0.90)	0.630
**Fibre (g/day)**	−0.06 (−0.09, −0.03)	0.001	−0.06 (−0.11, −0.004)	**0.034**
**CHO (%E)**	0.01 (−0.03, 0.05)	0.687	··	··
**Protein (%E**	0.05 (−0.02, 0.132)	0.154	··	··
**Total fat (%E)**	−0.03 (−0.07, 0.02)	0.197	−0.04 (−0.09, 0.008)	0.097
**Trans-fat (%E)**	0.40 (−0.76, 1.56)	0.492	··	··
**Saturated and trans-fat (%E)**	0.02 (−0.10, 0.06)	0.628	··	··
**Monosaturated fat (%E)**	−0.04 (−0.18, 0.10)	0.545	··	··
**Polyunsaturated fat (%E)**	−0.14 (−0.31, 0.27)	0.100	··	··
**Alcohol (%E)**	0.01 (−0.06, 0.08)	0.820	−0.03 (−0.09, 0.04)	0.460
**DF (%E)**	0.007 (−0.01, 0.03)	0.437	−0.006 (−0.03,0.02)	0.673
**SSBs (%E)**	0.04 (−0.05, 0.13)	0.340	··	··
**Added sugar intake (%E)**	0.06 (−0.01, 0.12)	0.075	··	··
**Total energy (MJ)**	−0.11 (−0.28, 0.04)	0.144	0.15 (0.09, 0.39)	0.209
**Total PA (minutes/wk) cont ^1^**	−0.003 (−0.004, −0.001)	0.005	−0.002 (−0.004, −0.001)	0.013
**Total PA (minutes/wk) ^1^**				
Did not meet recommended guideline (ref.)				
Met recommended guidelines	−0.96 (−1.95, 0.03)	0.057	··	··

Data were analysed using linear regression with data presented from univariable and multivariable linear regression analyses. Collinear diet variables were excluded (protein correlate with meat and alternatives, carbohydrate with alcohol, monosaturated fat, polyunsaturated fat, trans-fat saturated fat + trans-fat correlated with total fat, added sugar correlated with SSBs). β, beta-coefficient; CHO, carbohydrate; DF, discretionary foods; MJ, megajoules; MVPA, moderate-vigorous physical activity; PA, physical activity; SEIFA, socio-economic index of disadvantage for areas; SSBs, sugar sweetening beverages, %E, percentage energy. ^1^ Total minutes of physical activity undertaken in last week (includes walking for transport + walking for fitness + Moderate + Vigorous time but do not include sessions). Used as continuous and dichotomized as whether physical activity last week met 150 min recommended guidelines. The difference in the number of variables included in the multivariable analysis for pre-pregnant and postpartum women is due to model selection strategies using backward elimination techniques.

**Table 6:** Sensitivity analysis of the associations between diet and physical activity and BMI after excluding implausible energy reporters among pre-pregnant and postpartum women.

	Pre-Pregnancy (n = 577) ^§^		Postpartum (n = 989) ^§^	
	Adjusted β (95% CI)	*p* Value	Adjusted β (95% CI)	*p* Value
**Age, year**	0.20 (0.13, 0.27)	**<0.001**	0.14 (0.08., 0.19)	**<0.001**
**Country of birth**				
Australia (ref.)				
Other English-speaking country	0.39 (−0.96, 1.74)	0.567	0.18 (−1.61, 1.97)	0.841
Others	−1.66 (−2.70, −0.62)	**0.002**	−0.68 (−2.11, 0.75)	0.154
**Remoteness**				
Major cities (ref.)				
Inner regional	0.80 (−90, 2.51)	0.350	0.03 (−1.11, 1.17)	0.952
Other	1.70 (−0.67, 4.07)	0.157	1.18 (−0.46, 2.82)	0.154
**Marital status**				
Not married (ref.)				
Married	−0.35 (−1.44, 0.74)	0.517	...	..
**Education**				
Bachelor/Graduate diploma (ref.)				
Certificates/Advanced diploma	−0.75 (−2.54, 1.05)	0.408	−0.68 (−1.86, 0.50)	0.254
No non-school qualification	−0.28 (−1.93, 1.36)	0.731	1.08 (−0.07, 2.22)	0.064
**Occupation**				
Clerical trade (ref.)				
Professional	−0.31 (−1.99, 1.36)	0.710	−0.83 (−2.74, 1.09)	0.392
Assoc. Professional	−0.35 (−1.92, 1.22)	0.659	−1.46 (−3.20, 0.29)	0.100
Other	−1.51 (−2.96, −0.06)	**0.041**	−0.69 (−2.71, 1.33)	0.498
**SEIFA**				
1st (highest disadvantage) (ref.)				
2nd quintile	0.18 (−2.15, 2.50)	0.880	−0.48 (−2.27, 1.30)	0.591
3rd quintile	−0.42 (−2.49, 1.64)	0.684	−1.13 (−3.04, 0.78)	0.241
4th quintile	−0.62 (−2.75, 1.51)	0.564	−1.77 (−3.66, 0.12)	0.066
5th quintile (least disadvantage)	−0.49 (−2.52, 1.54)	0.631	−1.49 (−3.10, 0.12)	0.68
**Smoking status**				
Never smoked (ref.)				
Current smoker	−1.55 (−2.95, −0.16)	0.030	0.33 (−0.96, 1.62)	0.612
Ex-smoker	0.47 (−1.12, 2.06)	0.558	0.74 (−0.56, 2.04)	0.262
**Self-assessed health**				
Fair/poor (ref.)				
Excellent/very good/good	−2.22 (−5.09, 0.64)	0.125	−1.04 (−2.66, 0.58	0.203
**Dieting**				
Not currently on diet (ref)				
Currently on diet	2.28 (0.39, 4.17)	**0.019**	3.24 (1.79, 4.71)	**<0.001**
**Vegetables, legumes/beans (binary)**				
<5 serves/day (ref.)				
≥5 serves/day	−1.23 (−2.46, 0.003)	0.051	0.63 (−0.72, 1.99)	0.354
**Fruit (binary)**				
<2 serves/day (ref.)				
≥2 serves	0.86 (−0.31, 2.04)	0.146	−0.74 (−2.07, 0.59)	0.270
**Grain (cereal) foods (binary)**				
<6 serves (ref.)				
≥6 serves/day	1.44 (−0.21,3.09)	0.086	..	…
**Whole grain (serve, categorical)**				
<1 serves/day (ref.)				
≥1 to <2 serves/day	0.48 (−1.07, 2.03)	0.537	−1.10 (−2.17, −0.03)	0.044
≥2 to <3 serves/day	−0.58 (−2.44, 1.28)	0.537	−0.22 (−1.59, 1.15)	0.748
≥3 serves/day	−1.11 (−2.42, 0.20)	0.096	−0.78 (−2.35, 0.79)	0.324
**Milk and/or alternatives (binary)**				
<2.5 serves/day (ref.)				
≥2.5 serves/day	..	..	−0.26 (−1.38, 0.85)	0.641
**Meats and alternatives (binary)**				
<2.5 serves/day (ref.)				
≥2.5 serves/day	1.09 (−0.08, 2.25)	0.067	−0.66 (−1.88, 0.57)	0.287
**Fibre (g/day)**	..	…	−0.04 (−0.09, 0.01)	0.144
**Total fat (%E)**	−0.01 (−0.09, 0.07)	0.724	−0.04 (−0.09, 0.02)	0.211
**Alcohol (%E)**	−0.04 (−0.11, 0.03)	0.231	−0.01 (−0.08, 0.06)	0.737
**DF (%E)**	0.03 (−0.01, 0.07)	0.153	−0.0003 (−0.03, 0.03)	0.865
**SSBs (%E)**	0.06 (−0.06, 0.17)	0.303	..	..
**Total energy (MJ)**	−0.01 (−0.23, 0.21)	0.927	0.33 (0.09, 0.58)	**0.007**
**Total PA (minutes/wk) cont ^1^.**	−0.001(−0.003, 0.001)	0.208	−0.002 (−0.004, −0.0001)	**0.041**

Data were analysed using linear regression. Only data from multivariable linear regression analyses are presented. β, beta-coefficient; DF, discretionary foods; MJ, megajoules; MVPA, moderate-vigorous physical activity; PA, physical activity; SEIFA, socio-economic index of disadvantage for areas; SSBs, sugar sweetened beverages, %E, percentage energy. ^§^ Variables included here are similar to variables that were included in the main analyses and were selected based on backward stepwise elimination processes. 1 Total minutes of physical activity undertaken in last week (includes walking for transport + walking for fitness + Moderate + Vigorous time but do not include sessions).

## Data Availability

Publicly available datasets were analysed in this study and can be requested from the Australian Bureau of Statistics: the National Nutrition and Physical Activity Survey 2011–2012 in de-identified format.

## Supplementary Materials

The following supporting information can be downloaded at: https://www.mdpi.com/article/10.3390/nu14132607/s1. Table S1: Definitions of reproductive life stages. Table S2: Core food components, servings and daily recommended intakes according to the Australian Dietary Guideline (ADG 2013). Table S3: Discretionary food groups (food flag) based on the food code assigned in the food classifications system based on Australian Bureau of Statistics. Table S4 a–g: Dietary intake and physical activity by sociodemographic characteristics in pre-pregnant and postpartum women.
