# Supplementary material for: The Profiling of Diet and Physical Activity in Reproductive Age Women and Their Association with Body Mass Index
Source: Nutrients. 2022 Jun 23;14(13):2607. doi: 10.3390/nu14132607 (PMC9268133; doi:10.3390/nu14132607)
Supplement: Supplementary file 1 [file nutrients-14-02607-s001.zip › nutrients-1720921-supplementary.pdf]

Supplementary Materials

Table S1. Definition of key reproductive life stages (pre-pregnancy, pregnancy and postpartum) for women aged 18-48 years.

Survey question	Responses	Re-Coding	Life stage category
**Life stage for women (LS)**	1. Have never menstruated<br>2. Currently pregnant<br>3. Currently breastfeeding<br>4. Currently experiencing menopause<br>5. Post menopause<br>6. None of these apply<br>9. Not applicable	If **LS**=1 or 6 or 9 AND if **HHTYP**=1 or 2 or 5 or 6	**Pre-pregnancy**
		If **LS**=2	**Pregnant**
**The number of children' in the household (Household type, 'HHTYP')**	1. Person living alone<br>2. Couple only)<br>3. Couple family with children<br>4. One parent family with children<br>5. Unrelated persons aged 15+ only)<br>6. All other households	If LS=3 or 6 or 9 AND if **HHTYP**=3 or 4	**Postpartum**

If LS=4 or 5 were excluded.
Reproductive aged women 18-48 were included. This is to exclude lower limit of perimenopause
HHTYP=Household type; LS=life stage

Table S2. Core foods components, servings and daily recommended intakes according to the Australian Dietary Guideline (ADG 2013)[1]

**Core food groups**	**Components and description**	**Standard serves**	**Minimum recommended number of serves per day[1]**	**Variables for regression analysis**
**1. Vegetables (serves/day)**	Vegetables and legumes/beans from non-discretionary sources including different types and colours, and legumes/beans	75 g (100-350 kJ) or: ½ cup cooked green or orange vegetables (for example, broccoli, spinach, carrots or pumpkin), ½ cup cooked dried or canned beans, peas or lentils, 1 cup green leafy or raw salad vegetables, 1 medium tomato.	Women 19–50 y: 5 serves<br>Pregnant women: 5 serves<br>Breastfeeding women: 7 ½ serves	**Univariable model**:<br>a.<br><1 serves<br>≥1 to <3 serves<br>≥3 to <5 serves<br>≥5 serves<br>b.<br><5 serves/day vs. ≥5 serves<br>**Multivariable**: <5 serves/day vs. ≥5 serves
**2. Fruit (serves/day)**	Fruits in the form of fresh, frozen, canned, dried or juices	150 g (350 kJ) or: 1 medium apple, banana, pear or orange, 2 small apricots, kiwi fruits or plums, 1 cup diced or canned fruit (no added sugar) Or only occasionally: ½ cup fruit juice (no added sugar), 30 g dried fruit	2 serves for women 19-50 y, pregnant and breastfeeding mothers.	**Univariable model**:<br>a.<br><1 serves<br>≥1 to <2 serves<br>≥2 to <3 serves<br>≥3 serves<br>b.<br><2 serves/day vs. ≥2 serves<br>**Multivariable:**<br><2 serves/day vs. ≥2 serves/day
**3. Grain (cereal) foods (serves/day)**	Grains (cereals) includes: breads; breakfast cereals; crispbreads; grains (e.g., wheat, rice, oats, quinoa, barley, buckwheat); and grain products (e.g., pasta, noodles, couscous, bulgur, semolina, polenta, popcorn, flour), excluding discretionary grain foods (e.g., cakes, pastries and biscuits).	A standard serve is (500 kJ) or: 1 slice (40 g) of bread; ½ cup (75-120 g) cooked rice, pasta, noodles, barley, buckwheat, semolina, polenta, bulgur or quinoa, ¼ cup (30 g) muesli, 3 (35 g) crispbreads.	Women 19–50 y: 6 serves<br>Pregnant women: 8½ serves<br>Breastfeeding women: 9 serves	**Univariable model**:<br>a.<br>Zero or none<br>>0 to <2 serves<br>≥2 to <4 serves<br>≥4 to <6 serves<br>b.<br><6 serves/day vs. ≥6 serves/day<br>**Multivariable:**

				<6 serves/day vs. ≥6 serves/day
**4.. Whole grains**	Whole grain foods can be made from milled whole grains as well as intact, cracked, flaked or puffed grains including whole grain or wholemeal breads, multigrain breads, whole grain breakfast cereals, whole grain crispbreads, wholemeal pasta and wholemeal couscous, dry rice or pasta.	A food is a 100% whole-grain food if the only grains it contains are whole grains. One way to meet the recommendation to make at least half of grains whole grains is to choose products with at least 50 percent of the total weight as whole-grain ingredient<br><br>e.g. 3 serves: a small bowl of muesli (1/4 cup) for breakfast (= 1 serve) +2 slices of grainy bread at lunch as a sandwich (= 2 serves). One slice of bread is equivalent t 28 gram, 1 grams is made up of grains.	The Grains and Legumes Nutrition Council (GLNC) recommend Australians aged 9 years and above to meet a whole grain Daily Target Intake (DTI) of 48 g[2].<br>The 2015 US Dietary guidelines recommend consume at least half of total grains as whole grains (≥3 servings per day)[3].	**Univariable model:**<br>a.<br>< 1 serves/day<br>≥1 to <2 serves/day<br>≥2 to <3 serves/day<br>≥3 serves/day<br>b.<br><48 g per day<br>≥48 g per day<br>c.<br><50% of grains vs. ≥50% pf grains intake day<br>**Multivariable:**<br>< 1 serves/day<br>≥1 to <2 serves/day<br>≥2 to <3 serves/day<br>≥3 serves/day
**5. Dairy products and alternatives (serves/day)**	Dairy products and alternatives, mostly reduced fat	A standard serve is (500-600 kJ) or: 1 cup (250 ml) fresh, UHT long life, reconstituted powdered milk or buttermilk, ½ cup (120 ml) evaporated milk, 2 slices (40 g) of hard cheese, such as cheddar and 3/4 cup yoghurt.	2.5 serves for 19–50 y, pregnant and breastfeeding women	**Univariable model**:<br>a.<br><0.5 serves<br>≥0.5 to <1.5 serves<br>≥1.5 to<2.5 serves<br>≥2.5 serves<br>b.<br><2.5 serves/day vs. ≥2.5 serves/day<br>**Multivariable:**<br><2.5 serves/day vs. ≥2.5 serves/day
**6. Meat and alternatives (serves/day)**	Lean meat and poultry, fish, eggs, nuts and seeds, and legumes/beans: Incorporates foods from animal, seafood including lean	A standard serve is (500-600 kJ): 65 g cooked lean red meats such as beef, lamb, veal, pork, goat or kangaroo	Women 19–50 y: 2.5 serves	**Univariable model**:<br>a.<br><0.5 serves

	meats and poultry, fish, eggs, and plant-based alternatives such as tofu, legumes/beans and nuts and seeds	(about 90-100 g raw), 80 g cooked lean poultry such as chicken or turkey (100 g raw), 2 large eggs (120 g), 1 cup (150 g) cooked or canned legumes/beans such as lentils, chick peas or split peas, 170 g tofu , 30 g nuts, seeds, peanut or almond butter or tahini or other nut or seed paste.	Pregnant: 3.5serves<br>Breastfeeding women: 2.5 serves	≥0.5 to <1.5 serves<br>≥1.5 to<2.5 serves<br>≥2.5 serves<br>b.<br><2.5 serves/day vs. ≥2.5 serves/day<br>**Multivariable:**<br><2.5 serves/day vs. ≥2.5 serves/day

Y, year

[1] NHMRC. Australian Dietary Guidelines, Canberra: National Health and Medical Research Council,. Availabe online: https://www.nhmrc.gov.au/adg (accessed on 11).

[2] Griffiths, T & Nestel, P (2006) Developing a target for daily wholegrain intake for Australians. Food Aust 58, 431–433.

[3] US Department of Health and Human Services. (2019). US Department of Agriculture. 2015–2020 dietary guidelines for Americans. December 2015.

Table S3. Discretionary food groups (food flag)[1] based on the food code assigned in the food classifications system based on Australian Bureau of Statistics

Discretionary food/beverages	Food codes with examples
**Cereal based products and dishes (Sweet biscuits, Cakes, muffins and slices, Pastries and Mixed dishes where cereal is the major ingredient)**	13101-06 Sweet biscuits, plain or flavoured including short bread varieties; Sweet biscuits, plain with fruit or nuts; 13301-06, 13308, 13309 Cakes and cake mixes, chocolate, Cakes and cake mixes, sponge, Cakes and cake mixes, other types, Muffins, cake type, and muffin mixes, Scones and rock cakes, with added cheese, chocolate or similar; 13401-06 Pastry, plain/unfilled, all types; Sweet pastry products, fruit and/or nut fillings; Sweet pastry products, egg or dairy based fillings
**Sweet biscuits**	13101-07 Sweet biscuits, plain or flavoured including short bread varieties, Sweet biscuits, Sweet biscuits, plain with fruit or nuts, Sweet biscuits, with jam, marshmallow or other sugar-based filling, chocolate-coated, chocolate chip,
**Potato crisps and other similar snacks (Potato snacks, Corn snacks and Other snacks)**	26101, 26102 Potato crisps, 26201 Corn chips; Popcorn, commercial, butter flavoured, salted; 26301 Extruded snacks;
**Processed meats**	18601-07 Bacon; Ham; Processed delicatessen meat, mammalian; Processed delicatessen meat, poultry; Processed meat, commercially sterile (includes canned meats)
**Savoury sauces and condiments (Gravies and savoury sauces, Pickles, chutneys and relishes, Salad dressings and Dips)**	23101-05, 23107- 23110 Gravies (prepared); Savoury sauces, not tomato based, commercial; Savoury sauces, dairy based, homemade; 23301-04 Mayonnaise and cream-style dressings, full fat; Mayonnaise and cream-style dressings, reduced or non-fat;
**Ice cream and ice blocks (cream, Frozen milk products)**	19301-06; Cream, regular and increased fat; Cream, reduced fat Cream substitute, artificial crea; 19501-08 Ice cream, tub varieties, fat content >10 g/100 g; Ice cream, individual bar, stick and cone varieties, fat content >10 g/100 g; Frozen yoghurts, all types
**Jam and lemon spreads, chocolate spreads, sauces**	27201-05 Jams and conserves, sugar sweetened; Jams and conserves, reduced sugar; Sweet spreads or sauces, chocolate/coffee flavoured; Sauces, sweet, fruit-based
**Chocolate and chocolate-based confectionery, Other confectionery**	28101-04 Chocolate (plain, unfilled varieties), Chocolate-based confectionery with nut fillings or additions, Carob or yoghurt and carob or yoghurt-based confectionery. 28401-05 Lollies and other confectionery, sugar sweetened, Lollies and other confectionery, intense sweetened;

**Fruit and vegetable juices, and drinks**	11307 Fruit drinks (ready to drink or made from concentrate), 11308 Vegetable drinks, 11309 Fruit drink, prepared from dry powder
**Sweet snack bars (Fruit, nut and seed-bars, Muesli or cereal style bars)**	28201-02 Fruit bar and fruit-based confectionery; Nut and seed-based confectionery; 28301-05 Muesli and cereal style bars, no fruit; Muesli and cereal style bars, with fruit and/or nuts; Muesli bar, with fruit or fruit paste filling, Snack bar, other
**Alcoholic beverages (Beers, Wines, Other alcoholic beverages)**	29101-02 Beers, > 3.5% alcohol; Beers, 1.15- 3.5% alcohol, reduced alcohol / light; 29201-05 Wines, red (including sparkling varieties and rose styles); Wines, white (including sparkling varieties); 29501-05 Cocktails and other mixed drinks; Pre-mixed drinks, cola- or energy-drink based; Pre-mixed drinks, other
**Sugar sweetened beverages (SSBs) and fruit juice**	
**Soft drinks, and flavoured mineral waters**	11501-06 Soft drinks, non-cola, intense sweetened, Soft drinks, cola, Flavoured mineral waters
**Electrolyte, energy and fortified drinks**	11601-04 Electrolyte drinks (sports drinks), Electrolyte drink bases (sport drink bases), Energy drinks and Energy drinks, intense sweetened

[1] Food codes presented at the sub-major (3-digit code) or minor (5-digit code). The list of foods and the 'discretionary' flag is available at (https://www.abs.gov.au/AUSSTATS/abs@.nsf/DetailsPage/4363.0.55.0012011-13?OpenDocument)

Table S4. Dietary intake and physical activity by socio-demographic characteristics in pre-pregnant and postpartum women.

a. **Vegetable**

	Pre-pregnant (n = 880)				Postpartum (n = 1495)			
	**Total N (%)**	**Met guideline (n = 137) n (%)**	**Did not meet guideline (n = 743) n (%)**	**P value**	**Total N (%)**	**Met guideline (n = 224) n (%)**	**Did not meet guideline (n = 1271) n (%)**	**P value**
**Age, mean (SD)**	32.4±8.22	31.08 ± 8.17	31.6 ± 8.45	0.657	35.1 ± 8.20	33.6 ± 8.6	33.6 ± 9.25	0.946
**Country of birth**								
Australia born	645 (64.8)	96 (61.9)	549 (65.3)	0.810	1113 (75.0)	152 (67.0)	961 (76.4)	0.067
Main English speaking born	80 (10.7)	12 (10.7)	68 (10.7)		145 (9.77)	27 (12.7)	118 (9.26)	
Others	155 (24.5)	29 (27.5)	126 (23.9)		237(15.3)	45 (20.3)	192 (14.4)	
**Remoteness**								
Major city	596 (79.4)	92 (80.0)	504 (79.3)	0.797	972 (79.4)	156 (80.0)	816 (79.3)	0.797
Inner regional	150 (14.1)	25 (14.7)	125 (14.0)		286 (14.1)	32 (14.7)	254 (14.0)	
Others	134 (6.50)	20 (5.30)	114 (6.73)		237 (6.50)	36 (5.30)	201(6.73)	
**Marital status**								
Married	313 (38.6)	43 (38.6)	270 (38.6)	0.988	880 (57.8)	135 (61.8)	745 (57.1)	0.376
Not married	567 (61.4	94 (61.4)	473 (61.4)		615 (42.2)	89 (38.2)	526 (42.9)	
**Education**								
Bachelor/Graduate diploma	362 (42.0)	68 (51.0)	294 (40.3)	0.196	447 (29.4)	94 (43.8)	353 (15.7)	**<0.001**
Certificates/Advanced diploma	276 (33.3)	39 (30.4)	237(33.8)		523 (35.7)	60 (27.2)	463 (27.2)	
No non-school qualification	232 (24.7)	28 (18.6)	204 (25.9)		514 (35.0)	68 (28.9)	446 (28.9)	
**Occupation**								
Professional	320 (34.2)	51 (36.1)	269 (33.8)	0.774	369 (22.0)	64 (25.3)	305 (21.5)	0.640
Assoc. Professional	296 (33.0)	45 (30.1)	251 (33.5)		474 (34.8)	62 (31.1)	412 (35.6)	
Clerical trade	135 (16.2)	21 (19.3)	114 (15.7)		177 (12.7)	25 (11.9)	152 (12.8)	
Other	129 (16.5)	20 (13.7)	109 (17.1)		475 (30.4)	73 (31.7)	402 (30.2)	
**SEIFA**								
1st (higher disadvantage) (ref.)	151 (15.0)	18 (10.8)	133 (15.8)	0.204	277 (18.6)	40 (14.8)	137 (19.4)	0.242
2nd quintile	162 (18.4)	28 (21.0)	134 (18.0)		274 (17.6)	38 (17.6)	236 (17.7)	
3rd quintile	176 (23.7)	24 (25.1)	152 (23.5)		316 (21.6)	44 (21.6)	272 (22.2)	
4th quintile	158 (20.5)	19 (13.7)	139 (21.8)		260 (16.9)	41 (16.9)	219 (16.7)	

5th quintile (most advantage)	233 (22.3)	48 (29.4)	185 (20.9)		368 (25.4)	61 (25.4)	307 (24.1)	

Data were analysed using chi-square test and independent Student's t-test to compare between groups as appropriate.
SEIFA, Socio-Economic Index of Disadvantage for Areas; SSBs, sugar sweetening beverage.

b. Fruit

	Pre-pregnancy (n = 880)				Postpartum (n = 1495)			
	**Total N (%)**	**Met guideline (n = 229) n (%)**	**Did not meet guideline (n = 651) n (%)**	**P value**	**Total N (%)**	**Met guideline (n = 380) n (%)**	**Did not meet guideline (n = 1115) n (%)**	**P value**
**Age, mean (SD)**	32.4 ± 8.22	31.02 ± 7.77	31.2 ± 8.37	0.821	35.1 ± 8.20	33.9 ± 9.33	33.5 ± 9.10	0.591
**Country of birth**								
Australia born	64 5(64.8)	156 (60.1)	489 (66.2)	0.358	1113 (75.0)	262 (68.9)	851 (77.0)	**0.028**
Main English speaking born	80 (10.7)	23 (10.0)	57 (11.0)		145 (9.77)	39 (10.1)	106 (9.68)	
Others	155 (24.5)	50 (22.8)	105 (22.8)		237 (15.3)	79 (21.0)	158 (13.3)	
**Remoteness**								
Major city	596 (79.4)	155 (78.3)	441 (79.8)	0.902	972 (72.6)	248 (75.9)	724 (71.6)	0.383
Inner regional	150 (14.1)	43 (14.9)	107 (13.8)		286 (18.2)	73 (15.9)	213 (19.0)	
Others	134 (6.50)	31 (6.80)	103 (6.40)		237(9.16)	59 (8.20)	178 (9.48)	
**Marital status**								
Married	313 (38.6)	86 (35.2)	227 (39.8)	0.380	880 (57.8)	243 (61.4)	637 (56.7)	0.249
Not married	567 (61.4	143 (64.8)	424 (60.2)		615 (42.2)	137 (38.6)	478 (43.4)	
**Education**								
Bachelor/Graduate diploma	362 (42.0)	114 (49.3)	248 (39.4)	0.082	447 (29.4)	154(40.0)	293 (25.9)	**0.001**
Certificates/Advanced diploma	276 (33.3)	62 (31.7)	214 (33.8)		523 (35.7)	119 (31.1)	404 (37.2)	
No non-school qualification	232 (24.7)	53 (19.0)	179 (26.7)		514 (35.0)	100 (28.8)	414 (37.0)	
**Occupation**								
Professional	320 (34.2)	101 (43.3)	219 (31.0)	0.110	369 (22.0)	121 (30.0)	248 (19.4)	**0.014**
Assoc. Professional	296 (33.0)	70 (30.5)	226 (33.9)		474 (34.8)	111(30.6)	363 (36.2)	
Clerical trade	135 (16.2)	29 (12.9)	106 (17.4)		177 (12.7)	43 (12.7)	134 (12.7)	
Other	129 (16.5)	29 (13.3)	100 (17.7)		475 (30.4)	105 (26.7)	370 (31.7)	
**SEIFA**								
1st (higher disadvantage) (ref.)	151 (15.0)	32 (13.3)	119 (15.6)	0.372	277 (18.6)	58 (14.7)	219 (20.0)	0.128

2nd quintile	162 (18.4)	36 (13.3)	126 (20.2)		274 (17.6)	54 (13.8)	220 (18.9)	
3rd quintile	176 (23.7)	49 (28.2)	127 (22.2)		316 (21.6)	73 (21.8)	243 (21.5)	
4th quintile	158 (20.5)	45 (22.2)	113 (19.9)		260 (16.9)	83 (20.4)	177 (15.7)	
5th quintile (most advantage)	233 (22.3)	67 (23.0)	166 (22.0)		368 (25.4)	112 (29.4)	256 (24.0)	

Data were analysed using chi-square test and independent Student's t-test to compare between groups as appropriate.

c. Grain (cereal) foods

	Pre-pregnancy (n = 880)				Postpartum (n = 1495)			
	**Total N (%)**	**Met guideline (n = 158) n (%)**	**Did not meet guideline (n = 722) n (%)**	**P value**	**Total N (%)**	**Met guideline (n = 293) n (%)**	**Did not meet guideline (n = 1202) n (%)**	**P value**
**Age, mean (SD)**	32.4 ± 8.22	31.21 ± 7.51	31.2 ± 8.39	0.956	35.1 ± 8.20	33.4 ± 9.52	33.6 ± 9.07	0.796
**Country of birth**								
Australia born	645 (64.7)	106 (57.6)	539 (66.5)	**0.033**	1113 (75.0)	184 (62.8)	929 (77.7)	**0.000**
Main English speaking born	80 (10.7)	10 (5.73)	70 (12.0)		145 (9.77)	34 (11.5)	111 (9.38)	
Others	155 (24.5)	42 (36.7)	113 (21.5)		237 (15.3)	75 (25.7)	162 (12.9)	
**Remoteness**								
Major city	596 (79.4)	117 (86.3)	479 (77.7)	0.130	972 (72.6)	196 (77.7)	776 (71.5)	0.105
Inner regional	150 (14.1)	25 (9.91)	125 (15.1)		286 (18.2)	45 (12.9)	241 (19.4)	
Others	134 (6.50)	16 (3.82)	118 (7.16)		237 (9.16)	52 (9.36)	185 (9.12)	
**Marital status**								
Married	313 (38.6)	56 (36.7)	257 (39.1)	0.664	880 (57.8)	197 (63.0)	683 (56.7)	0.194
Not married	567 (61.4	102 (63.3)	465 (60.9)		615 (42.2)	96 (37.0)	519 (43.3)	
**Education**								
Bachelor/Graduate diploma	362(42.0)	71 (49.0)	291 (40.3)	0.263	447 (29.4)	107(31.4)	340 (28.9)	0.327
Certificates/Advanced diploma	276 (33.3)	48 (28.9)	228 (34.4)		523 (35.7)	88 (30.1)	435 (36.9)	
No non-school qualification	232 (24.7)	38 (22.1)	194 (25.4)		514 (35.0)	97 (38.5)	417 (34.2)	
**Occupation**								
Professional	320 (34.2)	63 (35.2)	257 (33.9)	0.829	369 (22.0)	78 (20.6)	291 (22.4)	0.532
Assoc. Professional	296 (33.0)	50 (32.4)	246 (33.2)		474 (34.8)	76 (30.5)	398 (35.8)	
Clerical trade	135 (16.2)	23 (18.4)	112 (15.7)		177 (12.7)	29 (13.8)	148 (12.5)	
Other	12 9(16.5)	22 (13.9)	107 (17.2)		475 (30.4)	110 (35.2)	365 (29.4)	
**SEIFA**								

1st (higher disadvantage) (ref.)	151 (15.0)	29 (15.5)	122 (14.9)	0.607	277 (18.6)	46 (17.2)	231 (18.9)	0.621
2nd quintile	162 (18.4)	37 (22.2)	125 (17.5)		274 (17.6)	52 (16.9)	222 (17.8)	
3rd quintile	176 (23.7)	31 (25.6)	145 (23.3)		316 (21.6)	64 (20.3)	252 (21.9)	
4th quintile	158 (20.5)	20 (14.7)	138 (22.0)		260 (16.9)	46 (15.0)	214 (17.3)	
5th quintile (most advantage)	233 (22.3)	41 (22.0)	192 (22.4)		368 (25.4)	85 (24.2)	283 (24.2)	

Data were analysed using chi-square test and independent Student's t-test to compare between groups as appropriate.

d. Dairy/alternatives

	Pre-pregnancy (n = 880)				Postpartum (n = 1495)			
	**Total N (%)**	**Met guideline (n = 131) n (%)**	**Did not meet guideline (n = 749) n (%)**	**P value**	**Total N (%)**	**Met guideline (n = 251) n (%)**	**Did not meet guideline (n = 1244) n (%)**	**P value**
**Age, mean (SD)**	32.4 ± 8.22	30.80 ± 8.43	31.2 ± 8.18	0.709	35.1 ± 8.20	34.7 ± 9.55	33.4 ± 9.07	0.150
**Country of birth**								
Australia born	645 (64.7)	100 (67.9)	545 (64.3)	0.356	1113 (75.0)	206 (82.3)	907 (73.7)	0.109
Main English speaking born	80 (10.7)	12 (5.35)	68 (11.6)		145 (9.77)	16 (7.93)	129 (10.1)	
Others	155 (24.5)	19 (26.8)	136 (24.2)		237 (15.3)	29 (9.78)	208 (16.2)	
**Remoteness**								
Major city	596 (79.4)	82 (79.4)	514 (79.6)	0.912	972 (72.6)	150 (65.8)	822 (73.8)	0.104
Inner regional	150 (14.1)	28 (14.1)	122 (13.9)		286 (18.2)	58 (20.9)	228 (17.7)	
Others	134 (6.50)	21 (6.50)	113 (6.48)		237 (9.16)	43 (13.3)	194 (8.44)	
**Marital status**								
Married	313 (38.6)	49 (41.3)	264 (38.2)	0.664	880 (57.8)	156 (64.6)	724 (56.7)	0.075
Not married	567 (61.4	82 (58.7)	485 (61.8)		615 (42.2)	95 (35.4)	520 (43.3)	
**Education**								
Bachelor/Graduate diploma	362 (42.0)	63 (53.7)	299 (40.2)	0.121	447 (29.4)	78 (31.9)	369 (28.9)	0.639
Certificates/Advanced diploma	276 (33.3)	41 (27.5)	235 (34.1)		523 (35.7)	88 (36.7)	435 (35.5)	
No non-school qualification	232 (24.7)	25 (18.7)	207 (25.6)		514 (35.0)	81 (31.4)	433 (35.6)	
**Occupation**								
Professional	320 (34.2)	58 (42.8)	262 (32.8)	0.331	369 (22.0)	67 (25.1)	302 (21.5)	0.436
Assoc. Professional	296 (33.0)	41 (33.6)	255 (33.0)		474 (34.8)	82 (38.7)	392 (34.2)	
Clerical trade	135 (16.2)	15 (12.2)	120 (16.9)		177 (12.7)	23 (11.0)	154 (13.0)	

Other	129 (16.5)	17 (11.3)	112 (17.3)		475 (30.4)	79 (25.2)	396 (31.4)	
**SEIFA**								
1st (higher disadvantage) (ref.)	151 (15.0)	19 (12.1)	132 (15.5)	0.463	277 (18.6)	40 (15.7)	237 (19.1)	0.716
2nd quintile	162 (18.4)	23 (13.4)	139 (19.2)		274 (17.6)	43 (17.8)	231 (17.6)	
3rd quintile	176 (23.7)	22 (26.2)	154 (23.4)		316 (21.6)	60 (23.2)	256 (21.3)	
4th quintile	158 (20.5)	29 (26.9)	129 (19.5)		260 (16.9)	40 (14.7)	220 (17.3)	
5th quintile (most advantage)	233 (22.3)	38 (21.4)	195 (22.4)		368 (25.4)	68 (28.7)	300 (24.8)	

Data were analysed using chi-square test and independent Student's t-test to compare between groups as appropriate.

e. Lean meat/alternatives

	Pre-pregnancy (n = 880)				Postpartum (n = 1495)			
	Total N (%)	Met guideline (n = 206) n (%)	Did not meet guideline (n = 674) n (%)	P value	Total N (%)	Met guideline (n = 331) n (%)	Did not meet guideline (n = 1164) n (%)	P value
**Age, mean (SD)**	32.4 ± 8.22	31.6 ± 8.28	31.1 ± 8.20	0.601	35.1 ± 8.20	33.8 ± 9.29	33.5 ± 9.12	0.819
**Country of birth**								
Australia born	645 (64.7)	140 (60.3)	505 (66.0)	0.593	1113 (75.0)	235 (71.9)	878 (75.9)	0.569
Main English speaking born	80 (10.7)	24 (12.3)	56 (10.3)		145 (9.77)	35 (10.9)	110 (9.44)	
Others	155 (24.5)	42 (27.4)	113 (23.7)		237 (15.3)	61 (17.2)	176 (14.7)	
**Remoteness**								
Major city	596 (79.4)	143(78.3)	453 (79.7)	0.468	972 (72.6)	210 (71.0)	762 (73.1)	0.627
Inner regional	150 (14.1)	33 (16.7)	117 (13.3)		286 (18.2)	56 (18.1)	230 (18.2)	
Others	134 (6.50)	30 (4.98)	104 (6.93)		237 (9.16)	65 (10.9)	172 (8.66)	
**Marital status**								
Married	313 (38.6)	81 (39.8)	232 (38.3)	0.719	880 (57.8)	195 (54.9)	685 (58.7)	0.382
Not married	567 (61.4	125 (60.2)	442 (61.7)		615 (42.2)	136 (45.1)	479 (41.3)	
**Education**								
Bachelor/Graduate diploma	362 (42.0)	86 (43.4)	276 (41.6)	0.913	447 (29.4)	111 (34.1)	336 (28.0)	0.283
Certificates/Advanced diploma	276 (33.3)	66 (31.6)	210 (33.8)		523 (35.7)	113 (34.4)	410 (36.0)	
No non-school qualification	232 (24.7)	53 (25.1)	179 (24.6)		514 (35.0)	105 (31.5)	409 (36.0)	
**Occupation**								
Professional	320 (34.2)	66 (32.6)	254 (34.6)	0.931	369 (22.0)	82 (20.8)	287 (22.4)	0.411
Assoc. Professional	296 (33.0)	76 (32.6)	220 (33.2)		474 (34.8)	106 (32.4)	368 (35.5)	

Clerical trade	135 (16.2)	35 (18.1)	100 (15.7)		177 (12.7)	42 (16.3)	135 (11.7)	
Other	129 (16.5)	29 (16.7)	100 (16.5)		475 (30.4)	101 (30.5)	374 (30.4)	
**SEIFA**								
1st (higher disadvantage) (ref.)	151 (15.0)	28 (15.7)	123 (14.9)	0.670	277 (18.6)	52 (17.2)	225 (19.0)	0.982
2nd quintile	162 (18.4)	40 (17.0)	122 (18.9)		274 (17.6)	63 (18.5)	211 (17.4)	
3rd quintile	176 (23.7)	39 (19.7)	137 (24.9)		316 (21.6)	70 (22.0)	246 (21.5)	
4th quintile	158 (20.5)	42 (21.2)	116 (20.3)		260 (16.9)	63 (17.2)	197 (16.8)	
5th quintile (most advantage)	233 (22.3)	57 (26.5)	176 (21.1)		368 (25.4)	83 (25.1)	285 (25.4)	

Data were analysed using chi-square test and independent Student's t-test to compare between groups as appropriate.

f. Discretionary food intake (serve)[1]

	Pre-pregnancy (n = 880)				Postpartum (n = 1495)			
	**Total N (%)**	**<2.5 servings (n = 565) n (%)**	**≥2.5 servings (n = 315) n (%)**	**P value**	**Total N (%)**	**<2.5 servings (n = 915) n (%)**	**≥2.5 servings (n = 580) n (%)**	**P value**
**Age, mean (SD)**	32.4 ± 8.22	31.1 ± 8.59	31.3 ± 7.59	0.834	35.1 ± 8.20	33.2 ± 9.02	34.2 ± 9.34	0.255
**Country of birth**								
Australia born	645 (64.7)	425 (66.0)	220 (62.7)	**0.017**	1113 (77.2)	713 (71.5)	400 (75.9)	**0.001**
Main English speaking born	80 (10.7)	52 (13.3)	28 (6.47)		145 (11.3)	98 (7.36)	47 (9.44)	
Others	155 (24.5)	88 (20.7)	67 (30.8)		237 (11.5)	104 (21.1)	133 (14.7)	
**Remoteness**								
Major city	596 (79.4)	382 (79.9)	214 (78.5)	0.335	972 (72.6)	574 (70.4)	398 (76.2)	0.104
Inner regional	150 (14.1)	105 (14.6)	45 (13.3)		286 (18.2)	197 (20.3)	89 (14.9)	
Others	134 (6.50)	78 (5.45)	56 (8.26)		237 (9.16)	144 (9.32)	93 (8.92)	
**Marital status**								
Married	313 (38.6)	197 (38.2)	116 (39.3)	0.779	880 (57.8)	530 (56.5)	350 (59.9)	0.345
Not married	567 (61.4	368 (61.8)	199 (60.7)		615 (42.2)	385 (43.5)	230 (40.1)	
**Education**								
Bachelor/Graduate diploma	362 (42.0)	224 (38.3)	138 (48.3)	0.120	447 (29.4)	260 (28.7)	187 (30.3)	0.181
Certificates/Advanced diploma	276 (33.3)	183 (35.0)	93 (30.3)		523 (35.7)	328 (38.3)	195 (31.6)	
No non-school qualification	232 (24.7)	154 (26.7)	78 (21.3)		514 (35.0)	321 (33.0)	193 (38.0)	
**Occupation**								
Professional	320 (34.2)	193 (31.0)	127 (39.4)	0.233	369 (22.0)	229 (23.8)	140 (19.3)	0.092

Assoc. Professional	296 (33.0)	200 (35.3)	96 (29.3)		474 (34.8)	292 (35.4)	182 (34.0)	
Clerical trade	135 (16.2)	90 (17.7)	45 (13.8)		177 (12.7)	118 (13.7)	59 (11.2)	
Other	129 (16.5)	82 (15.9)	47 (17.5)		475 (30.4)	276 (27.2)	199 (35.5)	
**SEIFA**								
1st (higher disadvantage) (ref.)	151 (15.0)	85 (13.1)	66 (18.2)	0.395	277 (18.6)	182 (19.8)	95 (16.7)	0.425
2nd quintile	162 (18.4)	107 (19.2)	55 (17.1)		274 (17.6)	163 (17.2)	111 (18.3)	
3rd quintile	176 (23.7)	123 (25.5)	53 (20.7)		316 (21.6)	192 (20.9)	124 (22.6)	
4th quintile	158 (20.5)	98 (19.7)	60 (22.0)		260 (16.9)	148 (15.5)	112 (19.0)	
5th quintile (most advantage)	233 (22.3)	152 (22.4)	81 (22.0)		368 (25.4)	230 (26.6)	138 (23.4)	

Data were analysed using chi-square test and independent Student's t-test to compare between groups as appropriate.

[1] Australian Dietary Guidelines recommend discretionary foods to be consumed in small amounts, from 0 to 2·5 servings for females per day, with one serving containing 600 kJ.

g. Physical activity[1]

	Pre-pregnancy (n = 874)				Postpartum (n = 1484)			
	**Total N (%)**	**Met guideline (n = 528) n (%)**	**Did not meet guideline (n = 346) n (%)**	**P value**	**Total N (%)**	**Met guideline (n = 732) n (%)**	**Did not meet guideline (n = 752) n (%)**	**P value**
**Age, mean (SD)**	32.4 ± 8.24	30.8 ± 8.04	31.7 ± 8.48	0.356	35.0 ± 8.21	33.5 ± 9.20	33.7 ± 9.15	0.728
**Country of birth**								
Australia born	646 (64.8)	395 (67.3)	247 (61.2)	0.228	1107 (64.8)	560 (67.3)	547 (61.2)	0.228
Main English speaking born	78 (10.6)	50 (11.2)	28 (9.73)		142 (10.6)	79 (11.3)	63 (9.73)	
Others	154 (24.6)	83 (21.4)	71 (29.1)		235 (24.6)	93 (21.4)	142 (29.1)	
**Remoteness**								
Major city	594 (79.6)	373 (81.9)	221 (76.2)	0.241	967 (72.9)	492 (75.4)	475 (70.5)	0.246
Inner regional	147 (13.8)	83 (12.3)	64 (16.1)		283 (18.0)	131 (16.1)	152 (19.8)	
Others	133 (6.54)	72 (5.74)	61 (7.71)		234 (9.11)	109 (8.50)	125 (9.70)	
**Marital status**								
Married	311 (38.7)	183 (39.0)	128 (38.1)	0.842	874 (58.0)	430 (56.7)	444 (59.2)	0.451
Not married	563 (61.3)	345 (60.9)	218 (62.9)		610 (42.0)	302 (43.3)	308 (40.8)	
**Education**								
Bachelor/Graduate diploma	360 (42.2)	238 (47.0)	122 (35.1)	**0.016**	442 (29.2)	251 (33.6)	191 (15.7)	**0.012**
Certificates/Advanced diploma	273 (33.0)	165 (31.9)	108 (34.6)		519 (35.7)	234 (33.2)	285 (26.9)	
No non-school qualification	232 (24.9)	119 (21.1)	113 (30.3)		512 (35.1)	241 (33.3)	271 (57.4)	
**Occupation**								
Professional	318 (34.3)	208 (39.0)	110 (27.4)	0.075	365 (21.9)	200 (23.3)	165 (20.5)	0.159
Assoc. Professional	293 (32.9)	180 (31.8)	113 (34.6)		472 (35.0)	224 (36.5)	248 (33.6)	
Clerical trade	134 (16.1)	65 (14.0)	69 (19.2)		176 (12.8)	76 (10.0)	100 (15.3)	
Other	129 (16.6)	75 (15.1)	54 (18.9)		471 (30.4)	232 (30.2)	239 (30.6)	
**SEIFA quintile**								
Q1 (lowest)	149 (14.9)	72 (10.5)	77 (21.5)	**0.021**	273 (18.4)	114 (14.1)	159 (22.4)	**0.010**
Q2	161 (18.3)	97 (18.0)	64 (18.0)		273 (17.6)	126 (17.2)	147 (18.1)	
Q3	175 (23.8)	105 (24.5)	70 (22.7)		313 (21.6)	146 (20.5)	167 (22.5)	
Q4	157 (20.6)	97 (21.7)	60 (18.8)		258 (16.9)	148 (20.0)	110 (13.9)	
Q5(highest)	232 (22.4)	157 (25.2)	75 (18.2)		367 (25.6)	198 (28.2)	169 (23.1)	

Data were analysed using chi-square test and independent Student's t-test to compare between groups as appropriate. All proportion estimates are weighted estimates.

[1]Total minutes undertaken physical activity in last week (includes walking for transport + walking for fitness + Moderate + Vigorous time but without sessions). 'Met physical activity guideline', involves completing at least 150 minutes of physical activity in 7 days prior to interview.
